# Functional features of gene expression profiles differentiating gastrointestinal stromal tumours according to *KIT *mutations and expression

**DOI:** 10.1186/1471-2407-9-413

**Published:** 2009-11-27

**Authors:** Jerzy Ostrowski, Marcin Polkowski, Agnieszka Paziewska, Magdalena Skrzypczak, Krzysztof Goryca, Tymon Rubel, Katarzyna Kokoszyñska, Piotr Rutkowski, Zbigniew I Nowecki, Anna Jerzak Vel Dobosz, Dorota Jarosz, Wlodzimierz Ruka, Lucjan S Wyrwicz

**Affiliations:** 1 Department of Gastroenterology and Hepatology, Medical Center for Postgraduate Education, Warsaw, Poland; 2 Department of Gastroenterology, M Sklodowska-Curie Memorial Cancer Center and Institute of Oncology, Warsaw, Poland; 3 Department of Soft Tissue/Bone Sarcoma and Melanoma, M Sklodowska-Curie Memorial Cancer Center and Institute of Oncology, Warsaw, Poland; 4 Department of Molecular Biology, M Sklodowska-Curie Memorial Cancer Center and Institute of Oncology, Warsaw, Poland; 5 Department of Pathology, M Sklodowska-Curie Memorial Cancer Center and Institute of Oncology, Warsaw, Poland; 6 Department of Colorectal Cancer, M Sklodowska-Curie Memorial Cancer Center and Institute of Oncology, Warsaw, Poland; 7 Laboratory of Bioinformatics and Systems Biology M Sklodowska-Curie Memorial Cancer Center and Institute of Oncology, Warsaw, Poland; 8 Bioinfobank Institute, Poznan, Poland

## Abstract

**Background:**

Gastrointestinal stromal tumours (GISTs) represent a heterogeneous group of tumours of mesenchymal origin characterized by gain-of-function mutations in *KIT *or *PDGFRA *of the type III receptor tyrosine kinase family. Although mutations in either receptor are thought to drive an early oncogenic event through similar pathways, two previous studies reported the mutation-specific gene expression profiles. However, their further conclusions were rather discordant. To clarify the molecular characteristics of differentially expressed genes according to GIST receptor mutations, we combined microarray-based analysis with detailed functional annotations.

**Methods:**

Total RNA was isolated from 29 frozen gastric GISTs and processed for hybridization on GENECHIP^® ^HG-U133 Plus 2.0 microarrays (Affymetrix). *KIT *and *PDGFRA *were analyzed by sequencing, while related mRNA levels were analyzed by quantitative RT-PCR.

**Results:**

Fifteen and eleven tumours possessed mutations in *KIT *and *PDGFRA*, respectively; no mutation was found in three tumours. Gene expression analysis identified no discriminative profiles associated with clinical or pathological parameters, even though expression of hundreds of genes differentiated tumour receptor mutation and expression status. Functional features of genes differentially expressed between the two groups of GISTs suggested alterations in angiogenesis and G-protein-related and calcium signalling.

**Conclusion:**

Our study has identified novel molecular elements likely to be involved in receptor-dependent GIST development and allowed confirmation of previously published results. These elements may be potential therapeutic targets and novel markers of *KIT *mutation status.

## Background

Gastrointestinal stromal tumours (GISTs) arise from precursor cells shared with the interstitial cells of Cajal (ICC) [1,2] and encompass a group of heterogeneous neoplasms with different morphology, biologic behaviour, and genetic characteristics [3]. Histopathologically, GISTs are spindle-, epithelioid-, or mixed-cell tumours that usually develop in the wall of the gastrointestinal tract. GISTs can be classified as benign, borderline, or malignant tumours based on tumour size, mitotic index, and the invasion of surrounding tissues, and the majority of these tumours are clinically rather non-aggressive [3].

An early oncogenic event in the majority of GISTs is represented by gain-of-function mutations in either *KIT *or *platelet-derived growth factor receptor α (PDGFRA)*. Both KIT and PDGFRA belong to the subclass III family of receptor tyrosine kinases [4-6]. The receptor-activating mutations lead to self-phosphorylation of a kinase domain, with the subsequent activation of the JAK/STAT, PI3K/AKT, Ras/ERK, and PLC-γ intracellular pathways in a ligand-independent manner, transmitting mitogenic signals [7-17].

Although mutations in *KIT *and *PDGFRA *contribute to tumour development through similar pathways, they correlate with certain clinicopathological features and different responses to imatinib treatment [3]. Moreover, GISTs with different mutation types exhibit differential gene expression at the mRNA [18,19] and protein [20] levels.

Two previous studies [18,19] reported differences between the gene expression profile and pattern of oncogenic mutations. Both studies and additional analyses have confirmed the unexpected observation that a mutation of *KIT *or *PDGFRA *is associated with its increased expression at the mRNA level, but in terms of further conclusions Subramanian *et al*. [19] and Kang *et al*. [18] are rather discordant. Subramanian and colleagues selected 1875 of almost 28 000 genes or ESTs (expressed sequence tags) clusters represented on cDNA microarray that passed filtering criteria and used it for further analysis. Of these selected genes, 338 were differentially expressed between GISTs assigned to a *KIT *exon 11 mutation and other types of mutations. A total of 270 genes were differentially expressed between GISTs with a *PDGFRA *mutation and other GISTs. Notably, a *PDGFRA *mutation was observed in only 8 of 26 analyzed samples.

In contrast, Kang *et al*. [18], using high-density spotted oligonucleotide microarrays, selected 4693 out of 18 664 oligonucleotides representing LEADS™ clusters. Among this set of pre-selected genes, only 70 were differentially expressed between GISTs exhibiting different mutation status. Of these 70, Subramanian *et al*. found only 13 (19%) to be differentially expressed. Both groups reported that on the basis of gene expression signatures, GISTs harbouring different types of mutations could not to be perfectly distinguished. Moreover, because of the far-from-complete coverage of the human genome using the methods in these studies, only limited functional annotations were reported. Thus, although these two important studies have been published, major questions about GIST biology remain open.

To clarify the molecular characteristics of differentially expressed genes according to receptor status, we combined microarray-based data with functional annotations. We selected a model of gastric GIST to obtain a balanced set of tumours with mutations in either *KIT *or *PDGFRA *[21]. Significant differences in the molecular makeup of the two groups of gastric GISTs allowed the development of novel functional hypotheses regarding the transduction of intracellular signalling contributing to GIST development.

## Methods

### Patients

Between April 2005 and March 2008, 31 patients with a diagnosis of gastric GIST were prospectively selected for the study. All patients underwent tumour surgical resection through laparotomy in the Department of Soft Tissue/Bone Sarcoma and Melanoma, and the final diagnosis was obtained from the analysis of clinicopathological findings (Table 1). The study protocol was approved by the Cancer Center Bioethical Committee, and all patients signed informed consent before inclusion. The morphological diagnosis was confirmed by standard H&E staining and immunoreactivity to KIT (CD117) [22,23] on routinely formalin-fixed paraffin-embedded specimens. One to two tumour fragments, depending on tumour size, were snap frozen and stored at -72°C until use. Then, collections of cryostat sections were prepared from different parts of each tumour fragment. Upper and lower sections from each cryosection collection were evaluated by the pathologist (DJ) to control the relative content of non-tumour cells, and the remaining internal portion of the specimen was used in the study if it contained ≥95% tumour cells. Genomic DNA from tissue samples was purified using the DNeasy Tissue Kit, and total RNA was isolated using the RNeasy Mini Kit (both kits from Qiagen GmbH, Hilden, Germany).

**Table 1:** Patient clinical, pathological and molecular characteristics of analyzed GIST samples

No	Sex	Tumour histology	Tumour size (mm)	Mitotic activity	Group	Tumour grade	CD 117	Mutation
1		Spindle cell	15	0	1	Benign	1	K11:p.573 580dup

2	M	Spindle cell	18	2	1	Benign	0	K11:p.571 579(?)dup+ insL(dupins?)

3	F	Spindle cell	20	0	2	Benign	1	K11:p.664 676del

4	F	Epithelioid	25	2	2	Benign	1	P12:p.566_571delinsR

5	M	Spindle cell	28	1	2	Benign	1	K11:p.V559D

6	F	Spindle cell	30	2	2	Benign	1	K11: p.W557R

7	M	Spindle cell	30	3	2	Benign	1	K11: p.W557R; p.V559D

8	M	Spindle cell	35	0	2	Benign	0	P18:p.843 846del

9	M	Mixed	35	1	2	Benign	0	P18:p.D842V

10	F	Epithelioid	35	1	2	Benign	1	No mutation found

11	M	Mixed	40	1	2	Benign	0	P18: p.D842V

12	M	Spindle cell	40	1	2	Benign	1	K11:p.578_580dup

13	M	Epithelioid	50	2	2	Benign	1	P18: p.843 847delinsL

14	M	Spindle cell	53	5	3a	Benign	1	K11:p.V559D

15	M	Mixed	55	1	3a	Benign	1	K9:p.502 503dup

16	M	Spindle cell	60	4	3a	Benign	1	P18: p.D842V

17	M	Mixed	65	0	3a	Benign	1	P18:p.843 846del

18	M	Spindle cell	65	1	3a	Benign	1	K11: p.555 573del

19	M	Spindle cell	70	3	3a	Benign	1	K11:p.552_556del

20	M	Spindle cell	70	4	3a	Benign	1	K11: p.V560E

21	M	Mixed	80	2	3a	Benign	1	P18:p.D842V

22	F	Spindle cell	90	2	3a	Benign	1	P18:p.D842V

23	M	Mixed	90	5	3a	Benign	1	No mutation found

24	M	Mixed	95	1	3a	Benign	1	P12: p.566 571delinsK

25	F	Spindle cell	35	13	5	Borderline	1	K11:p.573 585dup+ins C

26	M	Mixed	40	7	5	Borderline	0	K11:p.555 556del homo

27	M	Spindle cell	55	7	6a	Malignant	0	No mutation found

28	M	Spindle cell	70	21	6a	Malignant	1	P18:p.D842V

29	M	Spindle cell	160	7	6b	Malignant	1	K11:p.556 563del

### KIT/PDGFRA genotyping and real-time RT-PCR analysis

DNA samples were tested for hot-spot mutation sites of *KIT *(exons 9, 11, 13, 14, and 17) and *PDGFRA *(exons 12, 14, and 18) by PCR amplification using primers and annealing temperatures as previously described [24,25]. PCR products were sequenced in two directions by fluorescent dideoxysequencing on an ABI Prism 3100 Sequence Detection System (Applied Biosystems, Foster City, CA).

Specific RNA concentrations were determined by real-time reverse transcriptase (RT)-PCR. Total tissue RNA was isolated with the RNeasy Mini Kit and QIAshredder columns (Qiagen GmbH, Hilden, Germany). Reverse transcription was performed with the SuperScript II Reverse Transcriptase reagent set (Invitrogen Co., Carlsbad, CA) according to the manufacturer's instructions. Quantitative evaluation of mRNA was performed on an ABI Prism 7000 Sequence Detection System with a 25-μl reaction mixture containing 12.5 μl 2× SYBR Green PCR Master Mix (Applied Biosystems, Foster City, CA), 5 μl cDNA, and 50 nM primers. Oligonucleotide primers for the analyzed *KIT/PDGFRA *transcripts were designed using Primer Express Software (Applied Biosystems, Foster City, CA) and are listed in Supplementary Table S1 (see Additional file 1). For each run, standard curves were generated for a primer set by serial dilution of pooled cDNA to counterbalance variations in PCR reaction efficiency. Melting curves were generated after each reaction to verify the melting temperature of the amplicon. In addition, the purity of the RT-PCR product was verified by agarose gel electrophoresis. To normalize nonspecific variations in real-time PCR, the normalization factor was calculated as the geometric mean of RNA concentrations of three control genes, *glyceraldehyde-3-phosphate dehydrogenase*, *ubiquitin C*, and *β-actin*.

### Gene expression analyses on microarrays

Gene expression profiling was carried out using Affymetrix oligonucleotide microarrays (GeneChip HG-U133plus2) as described previously [26].

To obtain gene expression measurements, the extraction of probe-level data was performed with a standard GC-RMA algorithm for background correction and summarization steps and least-variant set algorithm for normalization based on a least-variant set of probe sets. The calculations were performed using BioConductor (version 2.8.1) packages *gcrma *(version 2.14.1) and *FLUSH.LVS.bundle *(version 1.2.1, proportion = 0.6). To test the internal consistency of the data sets, we used principal component analysis, normalized unscaled standard error plots, and relative log expression plots.

The measured expression levels were log transformed (log_2_). For data filtration, we selected the probe sets exhibiting signal intensity above the threshold limit, which was established at the 95th percentile of the expression levels from Y-chromosome-linked probe set signals detectable in female samples. The low-expression (marginal) probe sets with levels below the threshold in at least 19 samples were rejected.

To establish gene expression profiles, differentially expressed probe sets in the pair-wise comparisons were identified using the Kruskal-Wallis test. The resulting *P *values were adjusted for testing of multiple hypotheses using the Benjamini-Hochberg procedure that controls a false discovery rate. The false discovery rate threshold was set to 0.1, and only probe sets exhibiting a minimum two-fold change in mean relative expression were included in the gene lists. Cluster analysis of probe sets exhibiting differential expression was also performed. The probe sets were divided into groups of distinct expression patterns by an evolutionary-driven k-means clustering algorithm with a distance metric derived from the Pearson correlation coefficient. Unsupervised average-linkage hierarchical clustering and PCA were used for a graphic summary and evaluation of relationships between samples.

Both statistical and clustering analyses were performed using a proprietary software working in the MATLAB (MathWorks) and Bioconductor (2.8.1) environments.

### Functional analyses of gene expression by Gene Ontology

Differentially expressed probe sets were annotated with Gene Ontology (GO) terms (GO.db version 2.2.5) using the Bioconductor packages *GOstats *(version 2.8.0, Affymetrix HG-U133 Plus 2.0 Array Annotation Data) and package *annotate *(version 1.20.1). The significance of differential representation of GO terms between specified lists of probe sets was determined by the hypergeometric test implemented in GOstats (version 2.8.0). *P *values returned by GOstats were corrected for testing of multiple hypotheses with the Benjamini-Hochberg method implemented in an R environment (version 2.8.1, The R Foundation for Statistical Computing; http://www.r-project.org). Adjusted *P *values of less than 0.1 were considered significant.

Models of KIT and PDGFRA (a and b subunits) signalling pathways were prepared on the basis of three databases: Biogrid (The Biological General Repository for Interaction Datasets) [27], HPRD (Human Protein Reference Database) [28], and BIND (Biomolecular Interaction Network Database) [29] and additional literature searches.

## Results

### KIT and PDGFRA mutation profiling

Gene expression profiles from 29 out of 31 primary gastric GISTs were selected for the molecular analysis. Two samples were rejected due to poor quality of extracted information after MAS5.0 testing of criteria suggested by the producer. Of the 29 cases, 24, 2, and 3 cases were classified as benign (very low or low risk), borderline (intermediate risk), and malignant (high risk), respectively (Table 1). Such a distribution is consistent with the general clinical picture of gastric GISTs [21]. Genotyping revealed that 15 tumours had *KIT *mutations (14 in exon 11 and one in exon 9), 11 tumours had *PDGFRA *mutations (9 in exon 18 and two in exon 12), and 3 were *KIT/PDGFRA *wild-type GISTs within the analyzed mutation hotspots (Table 1). A total of 23 tumours were CD117/KIT-positive on immunohistochemistry (IHC). Of these, 13 and 8 tumours had *KIT *or *PDGFRA *mutations, respectively. In addition, two wild-type tumours were also KIT-positive. Thus, expression of KIT in IHC did not correlate with receptor tyrosine kinase mutation status (Table 1).

### Microarray results and mutation status

**Figure 1:**
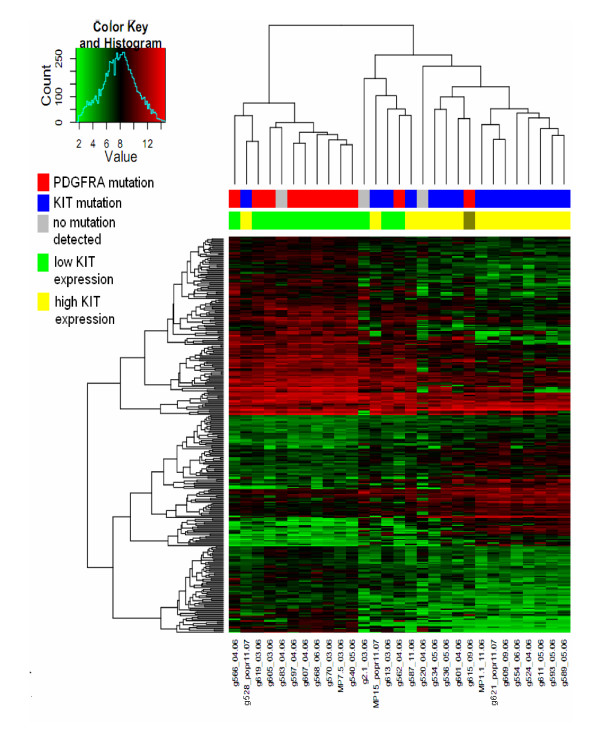
**Unsupervised hierarchical clustering for the selection of differentially expressed genes in GIST tumours according to *KIT *mutation**. Across the top, individual tumour samples are arrayed in a column (*upper*: blue - *KIT *mutation; red - *PDGFRA *mutation; gray- no mutation found; *lower*: green - low *KIT *expression/high *PDGFRA *expression; yellow- high *KIT *expression/low *PDGFRA *expression; dark gray- high *KIT*/*PDGFRA *expression); on the left side, 311 individual probe sets differentiating tumours in accordance with the mutation are shown in rows. The colour in each cell reflects the level of expression of the corresponding probe set in the corresponding array sample relative to its mean level of expression estimated for the entire set of samples. Red indicates expression levels greater than the mean, and green indicates lower than the mean.

From 54 675 probe sets of the Affymetrix HGU133plus2 microarray, 16 880 probe sets passed the filtering procedure. Among the probe sets with detectable expression levels, the non-parametric Kruskal-Wallis test revealed 970 (311 with FC>2; FC = fold change) probe sets differentially expressed between tumours with *KIT *and *PDGFRA *mutations. Of these 311 (FC>2), 109 probe sets (81 genes) were upregulated, and 202 probe sets (143 genes) were downregulated in samples from tumours harbouring *KIT *mutations compared to those with PDGFRA mutations. Supplementary Table S2 (see Additional file 1) gives the complete list of differentially expressed probe sets. As expected, unsupervised hierarchical clustering (Figure 1) of differentially expressed genes distinguished GIST samples according to the mutation status.

As reported previously [18,19], increased expression of *KIT *and *PDGFRA *follows their mutation status. To evaluate the impact of mutation status on expression of genes encoding both receptors, quantitative RT-PCR analysis was performed simultaneously on the same RNA samples used in the microarray analysis. An overall good correlation was observed between quantitative changes in *KIT *expression levels obtained by microarrays and quantitative RT-PCR (not shown). However, while the microarray signal intensity of *PDGFRA *probe sets was in most arrays slightly above the threshold limit, RT-PCR allowed for more reliable quantification of *PDGFRA *transcript levels.

**Figure 2:**
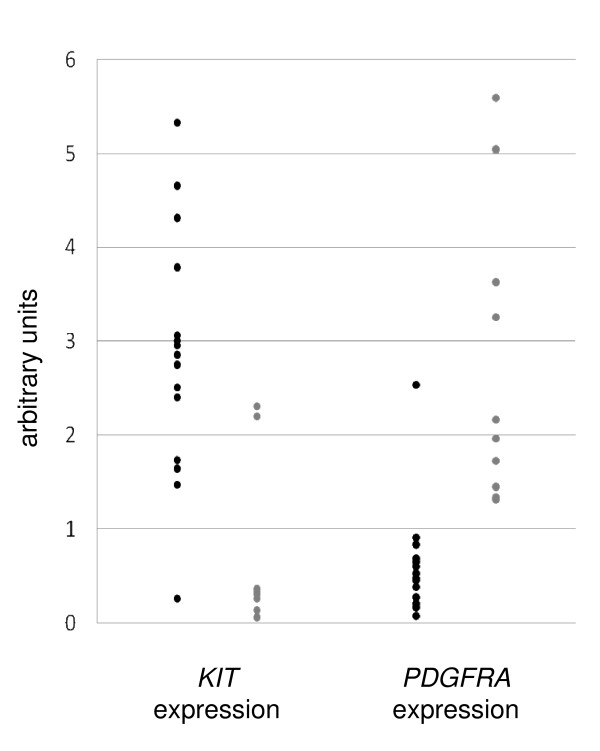
**Relative mRNA expression of *KIT *(left panel) and *PDGFRA *(right panel) in GIST tumours according to sample mutation status**. Black circles - KIT mutations; gray circles - PDGFRA mutations

Overexpression of *KIT *and *PDGFRA *was closely related to receptor mutation status. As shown in Figure 2, in 14 out of 15 GISTs with a *KIT *mutation and in 2 out of 10 GISTs with a *PDGFRA *mutation, the relative expression of *KIT *was 1.47 arbitrary units (a.u.) or greater, while in the remaining tumours, it was 0.37 a.u. or lower. In contrast, in nine tumours with a *PDGFRA *mutation and one tumour with a *KIT *mutation, the expression of *PDGFRA *was ≤1.32 a.u., while in the remaining GISTs with *KIT/PDGFRA *mutations, it was ≤0.84 a.u. One tumour with a *PDGFRA *mutation exhibited overexpression of both *KIT *and *PDGFRA*. Among receptor wild-type GISTs, the expression profile for one tumour was typical for tumours with *PDGFRA *mutations and was typical in two others for tumours with a *KIT *mutation. These findings suggest the presence of a potential additional region undergoing oncogenic mutations in both analyzed genes.

When selection of differentially expressed genes was performed according to the discriminating *KIT *transcript levels, the microarray data from the two groups of 15 and 13 tumour samples with either high or low *KIT *expression were included in analyses. At this point, 3116 (680 with the FC>2) probe sets indicated genes that were differentially expressed. (see Additional file 1: Supplementary Table S3). Of these, 253 probe sets (171 genes) were upregulated, and 427 probe sets (299 genes) were downregulated in tumours with overexpressed *KIT *compared to those with low expression. Again, unsupervised hierarchical clustering showed that gene expression patterns of the individual tumour samples from these two groups clustered together (see Additional file 2: Supplementary Figure S1).

No gene expression signature was found to be associated with tumour size, mitotic index, or risk category.

### Gene enrichment analyzes

Differential expression was then analyzed on the level of GO categories and KEGG (Kyoto Encyclopedia of Genes and Genomes) pathways. Functional features of the 311 and 680 genes that differentiated tumours according to receptor mutations and expression allowed selection of respectively nine and seven subcategories, as analyzed by their annotation to GO terms (see Additional file 1: Supplementary Table S4). Overrepresentation (estimated by the highest level of significance) was represented by *blood vessel development *(GO:0001568), *angiogenesis *(GO:0001525), *cell adhesion *(GO:0007155), and *G-protein coupled receptor protein signalling pathway *(GO:0007186). Terms that exhibited lower levels of statistical significance were represented by *elevation of cytosolic calcium ion concentration *(GO:0007204), *transmembrane receptor protein tyrosine kinase signalling pathway *(GO:0007169), and *cellular calcium ion homeostasis *(GO:0006874). In similar pair-wise comparisons performed for KEGG pathways, differentially regulated pathways were represented by *calcium signalling pathway *and *cytokine-cytokine receptor interaction*.

Interestingly, the expression of 7 genes annotated to synaptic transmission, 15 genes annotated to blood vessel development, and 20 genes annotated to G-protein signalling were at least 2-fold higher in tumours with low *KIT *expression compared to those with high *KIT *expression (see Additional file 1: Supplementary Table S5).

Because previous studies indicated that *KIT*-mutant and *PDGFRA*-mutant GISTs may have features associated with activation of downstream pathways like ERK1/2, AKT, p70/85S6K, STAT1/STAT3, and PI3K/mTOR [5,7-17], the lists of differentially expressed genes were compared with both lists of interacting partners of KIT and PDGFR, summarized in Supplementary Table S5 (see Additional file 1). According to our model of PDGFRA signalling pathways, the final data set contained 44 and 52 proteins interacting with KIT and PDGFR, respectively, 13 of which were common for both pathways (see Additional file 1: Supplementary Table S6). Within the list of differentially expressed genes according to the mutation status only 3 genes corresponding to PDGFRA interactome were found. None of KIT genes encoding KIT-signalling pathway proteins was found within this list.

To further test other pathways described by Corless *et al*. [5], we also analyzed genes from selected KEGG pathways involved in mitogenic signal transduction (mTOR, Jak-STAT, MAPK, TGF-beta, calcium signalling, vascular endothelial growth factor (VEGF) signalling). Those genes were compared with lists of differentially expressed genes depending on KIT/PDGFRA mutation status. Apart from protein kinase C (PKC)-alpha, no other genes from such lists were found among the differentially expressed genes (data not shown).

Among PKC isoforms analyzed in this study by microarray (alpha, beta isoform 1, variant 1, eta, iota, zeta) and quantitative RT-PCR (alpha, beta isoform 1, variant 1 and 2, delta, epsilon, theta) (data not shown), expression of PKC-alpha was significantly lower (FC = 0.17; *P *< 0.00016), while expression of PKC-theta was significantly higher (FC = 2.34; *P *< 0.00016) in tumours with *KIT *mutations compared to those with *PDGFRA *mutations and wild-type tumours.

## Discussion

GISTs express KIT, a 145-kD transmembrane glycoprotein that serves as the receptor for stem-cell factor [30,31]. KIT activates cellular signalling during embryogenesis [32,33] and is critical for the development of germ cells, hematopoietic progenitor cells, and mast cells [34]. Its function is closely related to that of other receptor tyrosine kinases for PDGF, macrophage colony stimulating factor, and FLT3 ligand [35]. Activating mutations in the *KIT *and *PDGFRA *genes result in ligand-independent activation of their receptor tyrosine kinase function, which may transmit early oncogenic signals in the majority of GISTs (Rutkowski *et al*., 2008).

Even though the biological consequences of *KIT *and *PDGFRA *mutations seem to be similar, our study has revealed hundreds of differentially expressed genes that group the tumours according to the receptor mutation status and receptor gene expression. However, although many of these genes may be involved in receptor-specific alterations of GIST intracellular signalling pathways, we identified no discriminative profiles of gene expression associated with clinical or pathological outcomes. Most of the discriminative genes were found to be upregulated in *PDGFRA*-mutated GISTS.

To further clarify if gene signatures that group GISTs according to *KIT/PDGFRA *mutation status, as described previously [18,19], may be defined also at the level of intracellular signalling pathways, we analyzed microarray data in the context of functional annotation.

Among terms and pathways in the current analysis with the highest overrepresentation in GISTs with mutated and/or overexpressed *PDGFRA *were "*blood vessel development*" and "*angiogenesis*". In fact, GISTs are highly vascularised tumours, and VEGF expression has been postulated to be a KIT-genotype-independent adverse prognostic indicator for early treatment failure and poor survival of GIST patients on imatinib therapy [36,37].

We also found that the functional features of genes differentially expressed between the two groups of GISTs were represented by the G-protein-coupled receptor protein signalling pathway (GO:0007186). The regulated secretion of transmitters and hormones, a characteristic event of neuroendocrine cells and tumours, is controlled by G-protein-coupled membrane receptors. Indirect evidence of neural or neuroendocrine phenotypes including high expression of this type of receptor have been described recently in GISTs [5].

As part of this study, we compiled lists of differentially expressed genes for comparison with lists of interacting partners of KIT and PDGFR. This analysis identified significantly lower expression of PKC-alpha and significantly higher expression of PKC-theta in tumours with *KIT *mutations compared to those with *PDGFRA *mutations or wild-type tumours. The PKC family consists of 10 related serine/threonine protein kinases, which are involved in regulation of cell proliferation, survival, and death. In addition, some are considered to be tumour promoters that may enhance multiple cellular oncogenic signalling pathways [38,39]. While the alpha, beta, epsilon, and atypical PKCs possess anti-apoptotic action, the delta and theta isoforms usually promote apoptosis [40]. Interestingly, PKC-theta has been selected previously as a sensitive marker of GISTs [41].

The enrichment analysis based on GO or similar annotations gives reliable results for sets of hundreds of genes, and such sets are likely to be identified with microarrays covering almost the whole transcriptome, as oligonucleotide microarrays which were used in this study. Experiments exploiting older technologies, like those used by Subramanian *et al*. [19] hold the middle ground between traditional single-gene techniques and high-throughput implementations and cannot provide sufficient data for systemic interpretation.

While the noise inherent in microarray technology often complicates the process of data interpretation, both the array quality and the choice of analytical processing methods have a major impact on differential expression analysis of microarray data [26]. Thus, stringent selection criteria are essential for identifying differentially expressed genes. In the case of a set of thousands of transcripts, *P *values and FC criteria are not sufficient if not coupled with an adequate *P *value correction method for simultaneous testing of multiple hypotheses (e.g., Bonferroni, Benjamini-Hochberg). An apparent lack of such a step in the study reported by Kang *et al*. (2005) is a likely reason for low concordance with other studies. With over 4000 genes tested, a *P *< 0.01 criterion leads to about 40 false positives (i.e., roughly 57% of a reported 70-gene data set). In the current work, we sought to address these issues through appropriate statistical adjustment for multiple hypotheses.

## Conclusion

To summarized, our study has identified novel molecular mechanisms likely to be involved in receptor-dependent GIST development and allowed confirmation of previously published results. These observations may be useful for the development of molecular markers that might predict which GIST patients will experience an adequate response to proposed therapy. However, before health-care professionals can see benefits from molecular diagnostics, a full understanding of the biological processes underlying GIST development is required.

## Competing interests

The authors declare that they have no competing interests.

## Authors' contributions

JO, MP - planned the analysis; AP, MS - performed gene expression studies; KG, TR, KK, LSW, JO - analyzed the results; MP, PR, ZIN, WR - provided clinical material; AJD - performed mutation analysis; DJ - provided histopathological expertise; JO, MP, LSW, KG, KK - wrote the manuscript. All authors read and approved the final manuscript.

## Pre-publication history

The pre-publication history for this paper can be accessed here:

http://www.biomedcentral.com/1471-2407/9/413/prepub

## Supplementary Material

Additional file 1 **Supplementary Tables S1-S6**. **Supplementary Table S1**. Primers used in this study. **Supplementary Table S2**. Probesets discriminating samples according to KIT mutation status (adjusted p.val < 0,1; FC>2). **Supplementary Table S3**. Probesets discriminating samples according to KIT transcript levels (adjusted p.val < 0,1; FC>2). **Supplementary Table S4**. A. Gene ontology terms overrepresented in probesets differentiating samples according to mutation status. B. Gene ontology terms overrepresented in probesets differentiating samples according to expression of KIT. **Supplementary Table S5**. Probesets with expression changed at least 2 fold between samples with low and high KIT expression annotated to selected GO terms: A. G-protein interaction (GO:0007186), B. synaptic transmission (GO:0007268), C. blood vessel development (GO:0001568). **Supplementary Table S6**. Interactomes of KIT and PDGFRA receptors assembled from literature and protein interaction databases (data sources: Bg - BioGrid, B - BOND database, H - HPRD, P - PubMed). Common proteins are highlighted in yellow. Click here for file

Additional file 2 **Supplementary Figure S1. Unsupervised hierarchical clustering for the selection of differentially expressed genes in GIST tumours according to *KIT *mutation**. Across the top, individual tumour samples are arrayed in a column (*upper*: green - low *KIT *expression/high *PDGFRA *expression; yellow- high *KIT *expression/low *PDGFRA *expression; dark gray- high *KIT*/*PDGFRA *expression; *lower*: blue - *KIT *mutation; red - *PDGFRA *mutation; gray- no mutation found); on the left side, 680 individual probe sets differentiating tumours in accordance with the KIT expression are shown in rows. The colour in each cell reflects the level of expression of the corresponding probe set in the corresponding array sample relative to its mean level of expression estimated for the entire set of samples. Red indicates expression levels greater than the mean, and green indicates lower than the mean. Click here for file
