# Supplementary material for: Functional features of gene expression profiles differentiating gastrointestinal stromal tumours according to KIT mutations and expression
Source: BMC Cancer. 2009 Nov 27;9:413. doi: 10.1186/1471-2407-9-413 (PMC2794290; doi:10.1186/1471-2407-9-413)
Supplement: Additional file 1 — Supplementary Tables S1-S6. Supplementary Table S1. Primers used in this study. Supplementary Table S2. Probesets discriminating samples according to KIT mutation status (adjusted p.val < 0,1; FC>2). Supplementary Table S3. Probesets discriminating samples according to KIT transcript levels (adjusted p.val < 0,1; FC>2). Supplementary Table S4. A. Gene ontology terms overrepresented in probesets differentiating samples according to mutation status. B. Gene ontology terms overrepresented in probesets differentiating samples according to expression of KIT. Supplementary Table S5. Probesets with expression changed at least 2 fold between samples with low and high KIT expression annotated to selected GO terms: A. G-protein interaction (GO:0007186), B. synaptic transmission (GO:0007268), C. blood vessel development (GO:0001568). Supplementary Table S6. Interactomes of KIT and PDGFRA receptors assembled from literature and protein interaction databases (data sources: Bg - BioGrid, B - BOND database, H - HPRD, P - PubMed). Common proteins are highlighted in yellow. [file 1471-2407-9-413-S1.DOC]

Supplementary table 1

Oligonucleotide sequences of primers used in PKC real-time PCR reactions.

| **Primer pairs used in real-time PCR reactions** | | |
| --- | --- | --- |
| Gene | Primer sequence | |
| *ACTB* | Forward: | 5’-AGCCTCGCCTTTGCCGA |
|  | Reverse: | 5’-GCGCGGCGATATCATCATC |
| *GAPDH* | Forward: | 5’-GAAGGTGAAGGTCGGAGTC |
|  | Reverse: | 5’-GAAGATGGTGATGGGATTTC |
| *KIT* | Forward: | 5’- GGCGACGAGATTAGGCTGTT |
|  | Reverse: | 5’- CATTCGTTTCATCCAGGATCTCA |
| *PDGFRA* | Forward: | 5’- GGCATTCTTTGCAATACTGCTTAA |
|  | Reverse: | 5’- CATCTGCCGATAGCACAGTGA |
| *PKCa* | Forward: | 5’-ACGTTCACAAGCAATGCGTC |
|  | Reverse: | 5’-TTAGGTAAATCCGCCCCCTC |
| *PKCb1* | Forward: | 5’-AACTCCATCGTTGAGCCTGG |
|  | Reverse: | 5’-CATGTGCACCGTGAATCCTG |
| *PKCb2* | Forward: | 5’-CCAAGAATGTGCTTTTAGAC |
|  | Reverse: | 5’-GGTAGCACCCAAGCTGGTTG |
| *PKCd* | Forward: | 5’-CCGACCATGTATCCTGAGTG |
|  | Reverse: | 5’-CCGCATTAGCACAATCTGGA |
| *PKCe* | Forward: | 5’-GTGTGACGACCACCACGTTC |
|  | Reverse: | 5’-GGGCCATACTCCAACTCCTG |
| *PKCt* | Forward: | 5’-CTGGCTGAGAGGTGCAGGA |
|  | Reverse: | 5’-TTAGCATTCGGCCTTGAGGT |
| *PKCg* | Forward: | 5’-ATGGACCCCAATGGTCTCTC |
|  | Reverse: | 5’-TCTGTTTCGTCAGGTTCCGA |
| *UBC* | Forward: | 5’-ATTTGGGTCGCGGTTCTTG |
|  | Reverse: | 5’-TGCCTTGACATTCTCGATGGT |

Supplementary table 2

Probesets discriminating samples according to *KIT* mutation status (adjusted p.val <0,1; FC>2)

| Probe | GenBank | Symbol | Description | adj,pval | fc |
| --- | --- | --- | --- | --- | --- |
| 221541_at | AL136861 | CRISPLD2 | cysteine-rich secretory protein LCCL domain containing 2 | 0.060 | 0.254 |
| 230250_at | AI670852 | PTPRB | protein tyrosine phosphatase, receptor type, B | 0.060 | 0.252 |
| 201920_at | NM_005415 | SLC20A1 | solute carrier family 20 (phosphate transporter), member 1 | 0.060 | 0.278 |
| 203414_at | NM_012329 | MMD | monocyte to macrophage differentiation-associated | 0.060 | 0.216 |
| 222165_x_at | AK022885 | C9orf16 | chromosome 9 open reading frame 16 | 0.060 | 0.394 |
| 244455_at | AI732637 | KCNT2 | potassium channel, subfamily T, member 2 | 0.060 | 2.241 |
| 204480_s_at | NM_024112 | C9orf16 | chromosome 9 open reading frame 16 | 0.060 | 0.344 |
| 218613_at | NM_018422 | PSD3 | pleckstrin and Sec7 domain containing 3 | 0.060 | 0.433 |
| 226390_at | AA628398 | STARD4 | StAR-related lipid transfer (START) domain containing 4 | 0.060 | 3.253 |
| 41047_at | AI885170 | C9orf16 | chromosome 9 open reading frame 16 | 0.060 | 0.378 |
| 201941_at | BE349147 | CPD | carboxypeptidase D | 0.060 | 0.468 |
| 213093_at | AI471375 | PRKCA | protein kinase C, alpha | 0.060 | 0.117 |
| 201681_s_at | AB011155 | DLG5 | discs, large homolog 5 (Drosophila) | 0.060 | 0.463 |
| 236262_at | AA025351 | MMRN2 | multimerin 2 | 0.060 | 0.341 |
| 204368_at | NM_005630 | SLCO2A1 | solute carrier organic anion transporter family, member 2A1 | 0.060 | 0.234 |
| 212518_at | AB011161 | PIP5K1C | phosphatidylinositol-4-phosphate 5-kinase, type I, gamma | 0.060 | 0.354 |
| 223382_s_at | AL136903 | ZNRF1 | zinc and ring finger 1 | 0.060 | 0.468 |
| 223595_at | AF247167 | TMEM133 | transmembrane protein 133 | 0.060 | 0.266 |
| 227443_at | AI972386 | C9orf150 | chromosome 9 open reading frame 150 | 0.060 | 2.279 |
| 228776_at | AA430014 | GJC1 | gap junction protein, gamma 1, 45kDa | 0.060 | 0.146 |
| 201369_s_at | NM_006887 | ZFP36L2 | zinc finger protein 36, C3H type-like 2 | 0.060 | 0.463 |
| 202084_s_at | NM_003003 | SEC14L1 | SEC14-like 1 (S. cerevisiae) | 0.060 | 0.451 |
| 204165_at | NM_003931 | WASF1 | WAS protein family, member 1 | 0.060 | 0.370 |
| 205902_at | AJ251016 | KCNN3 | potassium intermediate/small conductance calcium-activated channel, subfamily N, member 3 | 0.060 | 0.253 |
| 205903_s_at | NM_002249 | KCNN3 | potassium intermediate/small conductance calcium-activated channel, subfamily N, member 3 | 0.060 | 0.242 |
| 225171_at | BE644830 | ARHGAP18 | Rho GTPase activating protein 18 | 0.060 | 0.368 |
| 227197_at | AI989530 | SGEF | Src homology 3 domain-containing guanine nucleotide exchange factor | 0.060 | 0.251 |
| 228108_at | AW274846 |  |  | 0.060 | 0.310 |
| 229506_at | BF114646 |  |  | 0.060 | 0.356 |
| 209543_s_at | M81104 | CD34 | CD34 molecule | 0.060 | 3.048 |
| 203355_s_at | NM_015310 | PSD3 | pleckstrin and Sec7 domain containing 3 | 0.060 | 0.495 |
| 219091_s_at | NM_024756 | MMRN2 | multimerin 2 | 0.060 | 0.387 |
| 225173_at | BE501862 | ARHGAP18 | Rho GTPase activating protein 18 | 0.060 | 0.332 |
| 226028_at | AA156022 | ROBO4 | roundabout homolog 4, magic roundabout (Drosophila) | 0.060 | 0.323 |
| 230588_s_at | AA906142 | LOC285074 | hypothetical protein LOC285074 | 0.060 | 0.197 |
| 209082_s_at | AF018081 | COL18A1 | collagen, type XVIII, alpha 1 | 0.060 | 0.216 |
| 211958_at | R73554 | IGFBP5 | insulin-like growth factor binding protein 5 | 0.060 | 0.242 |
| 211959_at | AW007532 | IGFBP5 | insulin-like growth factor binding protein 5 | 0.060 | 0.434 |
| 216997_x_at | AL358975 | TLE4 | transducin-like enhancer of split 4 (E(sp1) homolog, Drosophila) | 0.060 | 3.580 |
| 223877_at | AF329839 | C1QTNF7 | C1q and tumor necrosis factor related protein 7 | 0.060 | 11.881 |
| 228457_at | AI590190 |  |  | 0.060 | 0.412 |
| 228485_s_at | AW165999 | SLC44A1 | solute carrier family 44, member 1 | 0.060 | 2.523 |
| 239598_s_at | AA789296 | LPCAT2 | lysophosphatidylcholine acyltransferase 2 | 0.060 | 6.194 |
| 244040_at | N47474 |  |  | 0.060 | 0.346 |
| 203632_s_at | NM_016235 | GPRC5B | G protein-coupled receptor, family C, group 5, member B | 0.060 | 0.330 |
| 208964_s_at | AL512760 | FADS1 | fatty acid desaturase 1 | 0.060 | 3.994 |
| 209369_at | M63310 | ANXA3 | annexin A3 | 0.060 | 2.335 |
| 213496_at | AW592563 | LPPR4 | plasticity related gene 1 | 0.060 | 0.055 |
| 218345_at | NM_018487 | TMEM176A | transmembrane protein 176A | 0.060 | 0.256 |
| 220753_s_at | NM_015974 | CRYL1 | crystallin, lambda 1 | 0.060 | 2.830 |
| 223235_s_at | AB014737 | SMOC2 | SPARC related modular calcium binding 2 | 0.060 | 0.241 |
| 225129_at | AW170571 | CPNE2 | copine II | 0.060 | 0.246 |
| 225166_at | AU158022 | ARHGAP18 | Rho GTPase activating protein 18 | 0.060 | 0.360 |
| 231773_at | BF002046 | ANGPTL1 | angiopoietin-like 1 | 0.060 | 0.073 |
| 231973_s_at | AK001223 | ANAPC1 | anaphase promoting complex subunit 1 | 0.060 | 0.316 |
| 235044_at | H06649 | CYYR1 | cysteine/tyrosine-rich 1 | 0.060 | 0.288 |
| 222108_at | AC004010 | AMIGO2 | adhesion molecule with Ig-like domain 2 | 0.060 | 6.144 |
| 230129_at | BF589448 | PSTK | phosphoseryl-tRNA kinase | 0.060 | 2.654 |
| 235527_at | U55983 | LOC284214 | hypothetical protein LOC284214 | 0.060 | 16.717 |
| 203723_at | NM_002221 | ITPKB | inositol 1,4,5-trisphosphate 3-kinase B | 0.060 | 0.330 |
| 204995_at | AL567411 | CDK5R1 | cyclin-dependent kinase 5, regulatory subunit 1 (p35) | 0.060 | 0.279 |
| 209081_s_at | NM_030582 | COL18A1 | collagen, type XVIII, alpha 1 | 0.060 | 0.170 |
| 212226_s_at | AA628586 | PPAP2B | phosphatidic acid phosphatase type 2B | 0.060 | 0.372 |
| 212345_s_at | BE675139 | CREB3L2 | cAMP responsive element binding protein 3-like 2 | 0.060 | 0.456 |
| 213358_at | AB018345 | KIAA0802 | KIAA0802 | 0.060 | 0.222 |
| 227325_at | AW172584 | LOC255783 | hypothetical protein LOC255783 | 0.060 | 0.337 |
| 200920_s_at | AL535380 | BTG1 | B-cell translocation gene 1, anti-proliferative | 0.060 | 0.441 |
| 202340_x_at | NM_002135 | NR4A1 | nuclear receptor subfamily 4, group A, member 1 | 0.060 | 0.261 |
| 205051_s_at | NM_000222 | KIT | v-kit Hardy-Zuckerman 4 feline sarcoma viral oncogene homolog | 0.060 | 2.316 |
| 205952_at | NM_002246 | KCNK3 | potassium channel, subfamily K, member 3 | 0.060 | 0.443 |
| 210381_s_at | BC000740 | CCKBR | cholecystokinin B receptor | 0.060 | 5.531 |
| 212230_at | AV725664 | PPAP2B | phosphatidic acid phosphatase type 2B | 0.060 | 0.359 |
| 212314_at | AB018289 | KIAA0746 | KIAA0746 protein | 0.060 | 0.209 |
| 212344_at | AW043713 | SULF1 | sulfatase 1 | 0.060 | 0.214 |
| 212353_at | AI479175 | SULF1 | sulfatase 1 | 0.060 | 0.229 |
| 213013_at | BG164295 | MAPK8IP1 | mitogen-activated protein kinase 8 interacting protein 1 | 0.060 | 0.351 |
| 222833_at | AU154202 | LPCAT2 | lysophosphatidylcholine acyltransferase 2 | 0.060 | 4.368 |
| 228665_at | AI458003 | CYYR1 | cysteine/tyrosine-rich 1 | 0.060 | 0.353 |
| 240890_at | AA041298 | LOC643733 | hypothetical LOC643733 | 0.060 | 2.997 |
| 243946_at | AI679149 | SMOC2 | SPARC related modular calcium binding 2 | 0.060 | 0.198 |
| 200878_at | AF052094 | EPAS1 | endothelial PAS domain protein 1 | 0.060 | 0.396 |
| 201508_at | NM_001552 | IGFBP4 | insulin-like growth factor binding protein 4 | 0.060 | 0.421 |
| 202218_s_at | NM_004265 | FADS2 | fatty acid desaturase 2 | 0.060 | 3.876 |
| 202883_s_at | T79584 | PPP2R1B | protein phosphatase 2 (formerly 2A), regulatory subunit A, beta isoform | 0.060 | 0.280 |
| 204948_s_at | NM_013409 | FST | follistatin | 0.060 | 19.551 |
| 206227_at | NM_003613 | CILP | cartilage intermediate layer protein, nucleotide pyrophosphohydrolase | 0.060 | 0.207 |
| 209355_s_at | AB000889 | PPAP2B | phosphatidic acid phosphatase type 2B | 0.060 | 0.350 |
| 215305_at | H79306 | PDGFRA | platelet-derived growth factor receptor, alpha polypeptide | 0.060 | 0.233 |
| 219025_at | NM_020404 | CD248 | CD248 molecule, endosialin | 0.060 | 0.252 |
| 219867_at | NM_024944 | CHODL | chondrolectin | 0.060 | 124.798 |
| 220532_s_at | NM_014020 | TMEM176B | transmembrane protein 176B | 0.060 | 0.326 |
| 227526_at | AU151222 | CDON | Cdon homolog (mouse) | 0.060 | 0.250 |
| 227889_at | AI765437 | LPCAT2 | lysophosphatidylcholine acyltransferase 2 | 0.060 | 3.727 |
| 235033_at | AL577823 | NPEPL1 | aminopeptidase-like 1 | 0.060 | 0.447 |
| 239118_at | BF513715 | KCNA2 | potassium voltage-gated channel, shaker-related subfamily, member 2 | 0.060 | 0.218 |
| 239349_at | BE856929 | C1QTNF7 | C1q and tumor necrosis factor related protein 7 | 0.060 | 8.649 |
| 208963_x_at | BG165833 | FADS1 | fatty acid desaturase 1 | 0.062 | 3.020 |
| 209581_at | BC001387 | HRASLS3 | HRAS-like suppressor 3 | 0.062 | 2.074 |
| 227415_at | BF109303 | DGKH | diacylglycerol kinase, eta | 0.062 | 3.707 |
| 228184_at | AK023679 | DISP1 | dispatched homolog 1 (Drosophila) | 0.062 | 2.304 |
| 202709_at | NM_002023 | FMOD | fibromodulin | 0.062 | 0.287 |
| 205651_x_at | NM_007023 | RAPGEF4 | Rap guanine nucleotide exchange factor (GEF) 4 | 0.062 | 0.190 |
| 212311_at | AA522514 | KIAA0746 | KIAA0746 protein | 0.062 | 0.190 |
| 214581_x_at | BE568134 | TNFRSF21 | tumor necrosis factor receptor superfamily, member 21 | 0.062 | 0.367 |
| 201939_at | NM_006622 | PLK2 | polo-like kinase 2 (Drosophila) | 0.062 | 0.278 |
| 202796_at | NM_007286 | SYNPO | synaptopodin | 0.062 | 0.270 |
| 202804_at | AI539710 | ABCC1 | ATP-binding cassette, sub-family C (CFTR/MRP), member 1 | 0.062 | 0.451 |
| 203780_at | AF275945 | MPZL2 | myelin protein zero-like 2 | 0.062 | 0.394 |
| 205112_at | NM_016341 | PLCE1 | phospholipase C, epsilon 1 | 0.062 | 2.941 |
| 208962_s_at | BE540552 | FADS1 | fatty acid desaturase 1 | 0.062 | 3.495 |
| 210512_s_at | AF022375 | VEGFA | vascular endothelial growth factor A | 0.062 | 0.333 |
| 212354_at | BE500977 | SULF1 | sulfatase 1 | 0.062 | 0.219 |
| 219557_s_at | NM_020645 | NRIP3 | nuclear receptor interacting protein 3 | 0.062 | 0.312 |
| 222256_s_at | AK000550 | JMJD7 | jumonji domain containing 7 | 0.062 | 2.021 |
| 225384_at | BF001267 | DOCK7 | dedicator of cytokinesis 7 | 0.062 | 2.167 |
| 226197_at | AW173504 |  |  | 0.062 | 9.180 |
| 227080_at | AW003092 | ZNF697 | zinc finger protein 697 | 0.062 | 2.875 |
| 228340_at | BE967118 | TLE3 | transducin-like enhancer of split 3 (E(sp1) homolog, Drosophila) | 0.062 | 0.383 |
| 228977_at | AI669535 | LOC729680 | hypothetical protein LOC729680 | 0.062 | 19.909 |
| 202112_at | NM_000552 | VWF | von Willebrand factor | 0.064 | 0.326 |
| 202884_s_at | NM_002716 | PPP2R1B | protein phosphatase 2 (formerly 2A), regulatory subunit A, beta isoform | 0.064 | 0.184 |
| 203662_s_at | NM_003275 | TMOD1 | tropomodulin 1 | 0.064 | 0.444 |
| 203934_at | NM_002253 | KDR | kinase insert domain receptor (a type III receptor tyrosine kinase) | 0.064 | 0.341 |
| 204223_at | NM_002725 | PRELP | proline/arginine-rich end leucine-rich repeat protein | 0.064 | 0.358 |
| 205227_at | NM_002182 | IL1RAP | interleukin 1 receptor accessory protein | 0.064 | 3.444 |
| 205501_at | AI143879 | PDE10A | phosphodiesterase 10A | 0.064 | 0.288 |
| 210605_s_at | BC003610 | MFGE8 | milk fat globule-EGF factor 8 protein | 0.064 | 0.373 |
| 211178_s_at | AF038602 | PSTPIP1 | proline-serine-threonine phosphatase interacting protein 1 | 0.064 | 21.047 |
| 212724_at | BG054844 | RND3 | Rho family GTPase 3 | 0.064 | 5.411 |
| 219527_at | NM_017898 | MOSC2 | MOCO sulphurase C-terminal domain containing 2 | 0.064 | 4.012 |
| 221529_s_at | AF326591 | PLVAP | plasmalemma vesicle associated protein | 0.064 | 0.323 |
| 222351_at | AW009884 | PPP2R1B | protein phosphatase 2 (formerly 2A), regulatory subunit A, beta isoform | 0.064 | 0.086 |
| 224339_s_at | AB056476 | ANGPTL1 | angiopoietin-like 1 | 0.064 | 0.070 |
| 224932_at | AI814909 | CHCHD10 | coiled-coil-helix-coiled-coil-helix domain containing 10 | 0.064 | 0.478 |
| 227417_at | AW057543 | MOSC2 | MOCO sulphurase C-terminal domain containing 2 | 0.064 | 4.583 |
| 237719_x_at | H05023 | RGS7BP | regulator of G-protein signaling 7 binding protein | 0.064 | 2.832 |
| 239657_x_at | AI341823 | FOXO6 | forkhead box protein O6 | 0.064 | 6.743 |
| 202016_at | NM_002402 | MEST | mesoderm specific transcript homolog (mouse) | 0.068 | 0.402 |
| 202886_s_at | M65254 | PPP2R1B | protein phosphatase 2 (formerly 2A), regulatory subunit A, beta isoform | 0.068 | 0.236 |
| 204955_at | NM_006307 | SRPX | sushi-repeat-containing protein, X-linked | 0.068 | 0.383 |
| 206201_s_at | NM_005924 | MEOX2 | mesenchyme homeobox 2 | 0.068 | 0.179 |
| 206444_at | NM_000924 | PDE1B | phosphodiesterase 1B, calmodulin-dependent | 0.068 | 0.212 |
| 212473_s_at | BE965029 | MICAL2 | microtubule associated monoxygenase, calponin and LIM domain containing 2 | 0.068 | 0.286 |
| 212770_at | AW873621 | TLE3 | transducin-like enhancer of split 3 (E(sp1) homolog, Drosophila) | 0.068 | 0.420 |
| 222862_s_at | BG169832 | AK5 | adenylate kinase 5 | 0.068 | 0.253 |
| 218086_at | NM_015392 | NPDC1 | neural proliferation, differentiation and control, 1 | 0.068 | 2.219 |
| 226192_at | T68445 |  |  | 0.068 | 7.378 |
| 229317_at | BG231980 | KPNA5 | karyopherin alpha 5 (importin alpha 6) | 0.068 | 2.073 |
| 203231_s_at | AW235612 | ATXN1 | ataxin 1 | 0.070 | 2.664 |
| 203895_at | AL535113 | PLCB4 | phospholipase C, beta 4 | 0.070 | 3.536 |
| 204677_at | NM_001795 | CDH5 | cadherin 5, type 2 (vascular endothelium) | 0.070 | 0.407 |
| 204872_at | NM_007005 | TLE4 | transducin-like enhancer of split 4 (E(sp1) homolog, Drosophila) | 0.070 | 2.403 |
| 209436_at | AB018305 | SPON1 | spondin 1, extracellular matrix protein | 0.070 | 0.319 |
| 211597_s_at | AB059408 | HOPX | HOP homeobox | 0.070 | 4.791 |
| 212282_at | BF038366 | TMEM97 | transmembrane protein 97 | 0.070 | 0.249 |
| 212472_at | BE965029 | MICAL2 | microtubule associated monoxygenase, calponin and LIM domain containing 2 | 0.070 | 0.254 |
| 213993_at | AI885290 | SPON1 | spondin 1, extracellular matrix protein | 0.070 | 0.251 |
| 221858_at | N34407 | TBC1D12 | TBC1 domain family, member 12 | 0.070 | 3.002 |
| 224657_at | AL034417 | ERRFI1 | ERBB receptor feedback inhibitor 1 | 0.070 | 0.466 |
| 225481_at | AL040051 | FRMD6 | FERM domain containing 6 | 0.070 | 0.459 |
| 228438_at | AI948599 | LOC100132891 | hypothetical protein LOC100132891 | 0.070 | 2.750 |
| 230715_at | AI138969 | ZNF518B | zinc finger protein 518B | 0.070 | 2.181 |
| 237833_s_at | BF062366 | SNCAIP | synuclein, alpha interacting protein | 0.070 | 6.586 |
| 37022_at | U41344 | PRELP | proline/arginine-rich end leucine-rich repeat protein | 0.070 | 0.453 |
| 205111_s_at | NM_016341 | PLCE1 | phospholipase C, epsilon 1 | 0.073 | 2.779 |
| 208851_s_at | AL161958 | THY1 | Thy-1 cell surface antigen | 0.073 | 0.474 |
| 209437_s_at | AB051390 | SPON1 | spondin 1, extracellular matrix protein | 0.073 | 0.279 |
| 211165_x_at | D31661 | EPHB2 | EPH receptor B2 | 0.073 | 0.230 |
| 211685_s_at | AF251061 | NCALD | neurocalcin delta | 0.073 | 0.409 |
| 211744_s_at | BC005930 | CD58 | CD58 molecule | 0.073 | 3.145 |
| 211818_s_at | U88712 | PDE4C | phosphodiesterase 4C, cAMP-specific (phosphodiesterase E1 dunce homolog, Drosophila) | 0.073 | 0.349 |
| 213010_at | AI088622 | PRKCDBP | protein kinase C, delta binding protein | 0.073 | 2.358 |
| 214043_at | BF062299 | PTPRD | protein tyrosine phosphatase, receptor type, D | 0.073 | 5.462 |
| 218832_x_at | NM_004041 | ARRB1 | arrestin, beta 1 | 0.073 | 2.018 |
| 218856_at | NM_016629 | TNFRSF21 | tumor necrosis factor receptor superfamily, member 21 | 0.073 | 0.412 |
| 219511_s_at | NM_005460 | SNCAIP | synuclein, alpha interacting protein | 0.073 | 10.468 |
| 225105_at | BF969397 | OCC-1 | overexpressed in colon carcinoma-1 | 0.073 | 2.372 |
| 225263_at | BC001196 | HS6ST1 | heparan sulfate 6-O-sulfotransferase 1 | 0.073 | 0.366 |
| 227198_at | AW085505 | AFF3 | AF4/FMR2 family, member 3 | 0.073 | 0.347 |
| 227915_at | AI872284 | ASB2 | ankyrin repeat and SOCS box-containing 2 | 0.073 | 0.213 |
| 235019_at | BE878495 | CPM | carboxypeptidase M | 0.073 | 0.456 |
| 240145_at | AW628059 |  |  | 0.073 | 3.025 |
| 241399_at | AI142028 | FAM19A2 | family with sequence similarity 19 (chemokine (C-C motif)-like), member A2 | 0.073 | 21.748 |
| 202464_s_at | NM_004566 | PFKFB3 | 6-phosphofructo-2-kinase/fructose-2,6-biphosphatase 3 | 0.075 | 0.470 |
| 202948_at | NM_000877 | IL1R1 | interleukin 1 receptor, type I | 0.075 | 0.404 |
| 206101_at | NM_001393 | ECM2 | extracellular matrix protein 2, female organ and adipocyte specific | 0.075 | 0.313 |
| 206791_s_at | BF511742 | PDE4C | phosphodiesterase 4C, cAMP-specific (phosphodiesterase E1 dunce homolog, Drosophila) | 0.075 | 0.366 |
| 213869_x_at | AA218868 | THY1 | Thy-1 cell surface antigen | 0.075 | 0.453 |
| 213994_s_at | AI885290 | SPON1 | spondin 1, extracellular matrix protein | 0.075 | 0.359 |
| 222033_s_at | AA058828 | FLT1 | fms-related tyrosine kinase 1 (vascular endothelial growth factor/vascular permeability factor receptor) | 0.075 | 0.385 |
| 224583_at | AL565621 | COTL1 | coactosin-like 1 (Dictyostelium) | 0.075 | 0.493 |
| 225664_at | AA788946 | COL12A1 | collagen, type XII, alpha 1 | 0.075 | 0.215 |
| 226497_s_at | AA149648 |  |  | 0.075 | 0.447 |
| 227404_s_at | AI459194 | EGR1 | early growth response 1 | 0.075 | 0.416 |
| 228127_at | BF513479 |  |  | 0.075 | 0.420 |
| 230087_at | AI823645 | PRIMA1 | proline rich membrane anchor 1 | 0.075 | 0.163 |
| 233116_at | U82695 |  |  | 0.075 | 0.322 |
| 235108_at | BG105700 |  |  | 0.075 | 0.397 |
| 236378_at | BF681360 | CIB4 | calcium and integrin binding family member 4 | 0.075 | 0.255 |
| 202794_at | NM_002194 | INPP1 | inositol polyphosphate-1-phosphatase | 0.075 | 2.559 |
| 203868_s_at | NM_001078 | VCAM1 | vascular cell adhesion molecule 1 | 0.075 | 2.206 |
| 208502_s_at | NM_002653 | PITX1 | paired-like homeodomain 1 | 0.075 | 4.916 |
| 221636_s_at | AL136931 | MOSC2 | MOCO sulphurase C-terminal domain containing 2 | 0.075 | 3.352 |
| 224530_s_at | AY029176 | KCNIP4 | Kv channel interacting protein 4 | 0.075 | 2.830 |
| 227948_at | AI949549 | FGD4 | FYVE, RhoGEF and PH domain containing 4 | 0.075 | 4.435 |
| 236783_at | AI732844 | KCNIP4 | Kv channel interacting protein 4 | 0.075 | 3.489 |
| 243931_at | R64696 |  |  | 0.075 | 2.846 |
| 1554239_s_at | BC033780 | ZADH2 | zinc binding alcohol dehydrogenase domain containing 2 | 0.078 | 2.345 |
| 1555997_s_at | BM128432 | IGFBP5 | insulin-like growth factor binding protein 5 | 0.078 | 0.376 |
| 200974_at | NM_001613 | ACTA2 | actin, alpha 2, smooth muscle, aorta | 0.078 | 0.405 |
| 202082_s_at | AV748469 | SEC14L1 | SEC14-like 1 (S. cerevisiae) | 0.078 | 0.447 |
| 203015_s_at | AW136988 | SSX2IP | synovial sarcoma, X breakpoint 2 interacting protein | 0.078 | 2.314 |
| 203424_s_at | AW157548 | IGFBP5 | insulin-like growth factor binding protein 5 | 0.078 | 0.318 |
| 203836_s_at | D84476 | MAP3K5 | mitogen-activated protein kinase kinase kinase 5 | 0.078 | 0.377 |
| 203896_s_at | NM_000933 | PLCB4 | phospholipase C, beta 4 | 0.078 | 3.518 |
| 205174_s_at | NM_012413 | QPCT | glutaminyl-peptide cyclotransferase | 0.078 | 0.206 |
| 205712_at | NM_002839 | PTPRD | protein tyrosine phosphatase, receptor type, D | 0.078 | 5.896 |
| 207732_s_at | NM_021120 | DLG3 | discs, large homolog 3 (neuroendocrine-dlg, Drosophila) | 0.078 | 2.077 |
| 208850_s_at | AL558479 | THY1 | Thy-1 cell surface antigen | 0.078 | 0.451 |
| 214767_s_at | AL551046 | HSPB6 | heat shock protein, alpha-crystallin-related, B6 | 0.078 | 0.362 |
| 216942_s_at | D28586 | CD58 | CD58 molecule | 0.078 | 3.122 |
| 221910_at | BF131965 | ETV1 | ets variant gene 1 | 0.078 | 2.057 |
| 223217_s_at | BE646573 | NFKBIZ | nuclear factor of kappa light polypeptide gene enhancer in B-cells inhibitor, zeta | 0.078 | 0.262 |
| 225464_at | N30138 | FRMD6 | FERM domain containing 6 | 0.078 | 0.428 |
| 226498_at | AA149648 |  |  | 0.078 | 0.404 |
| 226782_at | BF001919 | SLC25A30 | solute carrier family 25, member 30 | 0.078 | 2.755 |
| 232267_at | AL162032 | GPR133 | G protein-coupled receptor 133 | 0.078 | 0.398 |
| 235706_at | AW663908 | CPM | carboxypeptidase M | 0.078 | 0.481 |
| 238480_at | AI871745 |  |  | 0.078 | 0.410 |
| 241302_at | AI654048 |  |  | 0.078 | 3.597 |
| 201625_s_at | BE300521 | INSIG1 | insulin induced gene 1 | 0.081 | 2.709 |
| 202724_s_at | NM_002015 | FOXO1 | forkhead box O1 | 0.081 | 0.456 |
| 203426_s_at | M65062 | IGFBP5 | insulin-like growth factor binding protein 5 | 0.081 | 0.397 |
| 203661_s_at | BC002660 | TMOD1 | tropomodulin 1 | 0.081 | 0.467 |
| 205173_x_at | NM_001779 | CD58 | CD58 molecule | 0.081 | 2.812 |
| 205352_at | NM_005025 | SERPINI1 | serpin peptidase inhibitor, clade I (neuroserpin), member 1 | 0.081 | 0.351 |
| 206472_s_at | NM_005078 | TLE3 | transducin-like enhancer of split 3 (E(sp1) homolog, Drosophila) | 0.081 | 0.463 |
| 210198_s_at | BC002665 | PLP1 | proteolipid protein 1 | 0.081 | 0.065 |
| 213362_at | N73931 | PTPRD | protein tyrosine phosphatase, receptor type, D | 0.081 | 5.054 |
| 215617_at | AU145711 | LOC26010 | viral DNA polymerase-transactivated protein 6 | 0.081 | 3.676 |
| 217995_at | NM_021199 | SQRDL | sulfide quinone reductase-like (yeast) | 0.081 | 2.516 |
| 219197_s_at | AI424243 | SCUBE2 | signal peptide, CUB domain, EGF-like 2 | 0.081 | 0.370 |
| 219308_s_at | NM_012093 | AK5 | adenylate kinase 5 | 0.081 | 0.252 |
| 222900_at | AJ400877 |  |  | 0.081 | 0.290 |
| 224506_s_at | BC006362 | PPAPDC3 | phosphatidic acid phosphatase type 2 domain containing 3 | 0.081 | 0.448 |
| 225078_at | AV686514 | EMP2 | epithelial membrane protein 2 | 0.081 | 0.498 |
| 226433_at | BF056204 | RNF157 | ring finger protein 157 | 0.081 | 0.471 |
| 227550_at | AW242720 | LOC143381 | hypothetical protein LOC143381 | 0.081 | 0.397 |
| 227657_at | AA722069 | RNF150 | ring finger protein 150 | 0.081 | 3.507 |
| 228224_at | AA573140 | PRELP | proline/arginine-rich end leucine-rich repeat protein | 0.081 | 0.375 |
| 228399_at | AI569974 | OSR1 | odd-skipped related 1 (Drosophila) | 0.081 | 7.953 |
| 230645_at | BF110588 | FRMD3 | FERM domain containing 3 | 0.081 | 0.309 |
| 231478_at | AI051127 | PDE4C | phosphodiesterase 4C, cAMP-specific (phosphodiesterase E1 dunce homolog, Drosophila) | 0.081 | 0.356 |
| 236302_at | R40892 | PPM1E | protein phosphatase 1E (PP2C domain containing) | 0.081 | 0.391 |
| 236421_at | AI204272 | ANKRD45 | ankyrin repeat domain 45 | 0.081 | 2.341 |
| 201693_s_at | AV733950 | EGR1 | early growth response 1 | 0.086 | 0.309 |
| 203837_at | NM_005923 | MAP3K5 | mitogen-activated protein kinase kinase kinase 5 | 0.086 | 0.500 |
| 204011_at | NM_005842 | SPRY2 | sprouty homolog 2 (Drosophila) | 0.086 | 2.378 |
| 205593_s_at | NM_002606 | PDE9A | phosphodiesterase 9A | 0.086 | 0.490 |
| 209032_s_at | AF132811 | CADM1 | cell adhesion molecule 1 | 0.086 | 0.318 |
| 209281_s_at | M95541 | ATP2B1 | ATPase, Ca++ transporting, plasma membrane 1 | 0.086 | 0.467 |
| 209365_s_at | U65932 | ECM1 | extracellular matrix protein 1 | 0.086 | 0.364 |
| 211538_s_at | U56725 | HSPA2 | heat shock 70kDa protein 2 | 0.086 | 0.389 |
| 212171_x_at | H95344 | VEGFA | vascular endothelial growth factor A | 0.086 | 0.437 |
| 218806_s_at | AF118887 | VAV3 | vav 3 guanine nucleotide exchange factor | 0.086 | 0.444 |
| 219049_at | NM_018371 | CSGALNACT1 | chondroitin sulfate N-acetylgalactosaminyltransferase 1 | 0.086 | 0.433 |
| 220504_at | NM_007035 | KERA | keratocan | 0.086 | 0.307 |
| 221901_at | BF516072 | LL22NC03-75B3.6 | KIAA1644 protein | 0.086 | 0.253 |
| 222154_s_at | AK002064 | LOC26010 | viral DNA polymerase-transactivated protein 6 | 0.086 | 2.412 |
| 224836_at | AL109824 | TP53INP2 | tumor protein p53 inducible nuclear protein 2 | 0.086 | 0.497 |
| 225536_at | AL545105 | TMEM54 | transmembrane protein 54 | 0.086 | 2.209 |
| 226304_at | AA563621 | HSPB6 | heat shock protein, alpha-crystallin-related, B6 | 0.086 | 0.328 |
| 228618_at | AL040178 | PEAR1 | platelet endothelial aggregation receptor 1 | 0.086 | 3.003 |
| 230061_at | AW338625 | TM4SF18 | transmembrane 4 L six family member 18 | 0.086 | 0.368 |
| 203904_x_at | NM_002231 | CD82 | CD82 molecule | 0.091 | 0.372 |
| 204082_at | NM_006195 | PBX3 | pre-B-cell leukemia homeobox 3 | 0.091 | 0.406 |
| 206540_at | NM_024506 | GLB1L | galactosidase, beta 1-like | 0.091 | 0.323 |
| 208998_at | U94592 | UCP2 | uncoupling protein 2 (mitochondrial, proton carrier) | 0.091 | 0.391 |
| 219087_at | NM_017680 | ASPN | asporin | 0.091 | 0.415 |
| 219134_at | NM_022159 | ELTD1 | EGF, latrophilin and seven transmembrane domain containing 1 | 0.091 | 0.342 |
| 220006_at | NM_024768 | CCDC48 | coiled-coil domain containing 48 | 0.091 | 0.355 |
| 222885_at | AF205940 | EMCN | endomucin | 0.091 | 0.421 |
| 225328_at | N21643 |  |  | 0.091 | 0.394 |
| 225803_at | AW006123 | FBXO32 | F-box protein 32 | 0.091 | 0.454 |
| 226582_at | AL520272 | LOC400043 | hypothetical gene supported by BC009385 | 0.091 | 0.327 |
| 230228_at | W94546 | LOC284297 | hypothetical LOC284297 | 0.091 | 0.348 |
| 202747_s_at | NM_004867 | ITM2A | integral membrane protein 2A | 0.091 | 2.611 |
| 203474_at | NM_006633 | IQGAP2 | IQ motif containing GTPase activating protein 2 | 0.091 | 2.021 |
| 206389_s_at | NM_000921 | PDE3A | phosphodiesterase 3A, cGMP-inhibited | 0.091 | 2.353 |
| 210738_s_at | AF011390 | SLC4A4 | solute carrier family 4, sodium bicarbonate cotransporter, member 4 | 0.091 | 3.691 |
| 210809_s_at | D13665 | POSTN | periostin, osteoblast specific factor | 0.091 | 8.773 |
| 210831_s_at | L27489 | PTGER3 | prostaglandin E receptor 3 (subtype EP3) | 0.091 | 3.988 |
| 229024_at | BF056892 |  |  | 0.091 | 3.181 |
| 200862_at | NM_014762 | DHCR24 | 24-dehydrocholesterol reductase | 0.095 | 2.912 |
| 203425_s_at | NM_000599 | IGFBP5 | insulin-like growth factor binding protein 5 | 0.095 | 0.423 |
| 203908_at | NM_003759 | SLC4A4 | solute carrier family 4, sodium bicarbonate cotransporter, member 4 | 0.095 | 4.925 |
| 204803_s_at | NM_004165 | RRAD | Ras-related associated with diabetes | 0.095 | 2.345 |
| 205893_at | NM_014932 | NLGN1 | neuroligin 1 | 0.095 | 5.952 |
| 206510_at | AF332197 | SIX2 | SIX homeobox 2 | 0.095 | 0.374 |
| 206879_s_at | NM_013982 | NRG2 | neuregulin 2 | 0.095 | 2.044 |
| 209589_s_at | AF025304 | EPHB2 | EPH receptor B2 | 0.095 | 0.362 |
| 210374_x_at | D38300 | PTGER3 | prostaglandin E receptor 3 (subtype EP3) | 0.095 | 3.845 |
| 225033_at | AV721528 | LOC286167 | hypothetical LOC286167 | 0.095 | 0.365 |
| 228817_at | AI085361 | ALG9 | asparagine-linked glycosylation 9 homolog (S. cerevisiae, alpha- 1,2-mannosyltransferase) | 0.095 | 0.374 |
| 230258_at | AI277316 | GLIS3 | GLIS family zinc finger 3 | 0.095 | 0.425 |
| 230559_x_at | AI277617 | FGD4 | FYVE, RhoGEF and PH domain containing 4 | 0.095 | 4.018 |
| 230866_at | BE549540 | CYSLTR1 | cysteinyl leukotriene receptor 1 | 0.095 | 2.418 |
| 232136_s_at | AB051545 | CTTNBP2 | cortactin binding protein 2 | 0.095 | 2.100 |
| 235657_at | BF061389 |  |  | 0.095 | 2.387 |
| 235952_at | AA521504 |  |  | 0.095 | 2.766 |
| 236029_at | AI283093 | FAT3 | FAT tumor suppressor homolog 3 (Drosophila) | 0.095 | 5.154 |

Supplementary table 3

Probesets discriminating samples according to *KIT* transcript levels (adjusted p.val <0,1; FC>2)

| Probe | GenBank | Symbol | Description | adj,pval | fc |
| --- | --- | --- | --- | --- | --- |
| 201920_at | NM_005415 | SLC20A1 | solute carrier family 20 (phosphate transporter), member 1 | 0.016 | 4.279 |
| 227443_at | AI972386 | C9orf150 | chromosome 9 open reading frame 150 | 0.016 | 0.385 |
| 205051_s_at | NM_000222 | KIT | v-kit Hardy-Zuckerman 4 feline sarcoma viral oncogene homolog | 0.016 | 0.322 |
| 206444_at | NM_000924 | PDE1B | phosphodiesterase 1B, calmodulin-dependent | 0.016 | 28.325 |
| 218345_at | NM_018487 | TMEM176A | transmembrane protein 176A | 0.016 | 5.948 |
| 212518_at | AB011161 | PIP5K1C | phosphatidylinositol-4-phosphate 5-kinase, type I, gamma | 0.016 | 2.966 |
| 214298_x_at | AL568374 | SEPT6 | septin 6 | 0.016 | 2.059 |
| 221541_at | AL136861 | CRISPLD2 | cysteine-rich secretory protein LCCL domain containing 2 | 0.016 | 4.423 |
| 227197_at | AI989530 | SGEF | Src homology 3 domain-containing guanine nucleotide exchange factor | 0.016 | 4.762 |
| 205121_at | NM_000232 | SGCB | sarcoglycan, beta (43kDa dystrophin-associated glycoprotein) | 0.016 | 0.493 |
| 212414_s_at | D50918 | SEPT6 | septin 6 | 0.016 | 2.034 |
| 220532_s_at | NM_014020 | TMEM176B | transmembrane protein 176B | 0.016 | 4.236 |
| 237719_x_at | H05023 | RGS7BP | regulator of G-protein signaling 7 binding protein | 0.016 | 0.218 |
| 202259_s_at | NM_014887 | N4BP2L2 | NEDD4 binding protein 2-like 2 | 0.016 | 0.447 |
| 204480_s_at | NM_024112 | C9orf16 | chromosome 9 open reading frame 16 | 0.016 | 2.750 |
| 228184_at | AK023679 | DISP1 | dispatched homolog 1 (Drosophila) | 0.016 | 0.285 |
| 236262_at | AA025351 | MMRN2 | multimerin 2 | 0.016 | 3.339 |
| 203414_at | NM_012329 | MMD | monocyte to macrophage differentiation-associated | 0.016 | 4.585 |
| 209081_s_at | NM_030582 | COL18A1 | collagen, type XVIII, alpha 1 | 0.016 | 6.249 |
| 209369_at | M63310 | ANXA3 | annexin A3 | 0.016 | 0.346 |
| 211958_at | R73554 | IGFBP5 | insulin-like growth factor binding protein 5 | 0.016 | 5.621 |
| 226292_at | BF195709 | CAPN5 | calpain 5 | 0.016 | 2.194 |
| 48825_at | AA887083 | ING4 | inhibitor of growth family, member 4 | 0.016 | 0.490 |
| 200921_s_at | NM_001731 | BTG1 | B-cell translocation gene 1, anti-proliferative | 0.016 | 2.026 |
| 201508_at | NM_001552 | IGFBP4 | insulin-like growth factor binding protein 4 | 0.016 | 2.629 |
| 202218_s_at | NM_004265 | FADS2 | fatty acid desaturase 2 | 0.016 | 0.237 |
| 202709_at | NM_002023 | FMOD | fibromodulin | 0.016 | 3.451 |
| 203015_s_at | AW136988 | SSX2IP | synovial sarcoma, X breakpoint 2 interacting protein | 0.016 | 0.313 |
| 209082_s_at | AF018081 | COL18A1 | collagen, type XVIII, alpha 1 | 0.016 | 5.105 |
| 209392_at | L35594 | ENPP2 | ectonucleotide pyrophosphatase/phosphodiesterase 2 | 0.016 | 2.127 |
| 221910_at | BF131965 | ETV1 | ets variant gene 1 | 0.016 | 0.375 |
| 230129_at | BF589448 | PSTK | phosphoseryl-tRNA kinase | 0.016 | 0.351 |
| 202804_at | AI539710 | ABCC1 | ATP-binding cassette, sub-family C (CFTR/MRP), member 1 | 0.016 | 2.409 |
| 203320_at | NM_005475 | SH2B3 | SH2B adaptor protein 3 | 0.016 | 2.132 |
| 213391_at | AI669947 | DPY19L4 | dpy-19-like 4 (C. elegans) | 0.016 | 0.476 |
| 219091_s_at | NM_024756 | MMRN2 | multimerin 2 | 0.016 | 2.782 |
| 220753_s_at | NM_015974 | CRYL1 | crystallin, lambda 1 | 0.016 | 0.332 |
| 224791_at | AW513835 | DDEF1 | development and differentiation enhancing factor 1 | 0.016 | 2.000 |
| 225060_at | BF696304 | LRP11 | low density lipoprotein receptor-related protein 11 | 0.016 | 2.123 |
| 227618_at | AI250910 |  |  | 0.016 | 2.569 |
| 41047_at | AI885170 | C9orf16 | chromosome 9 open reading frame 16 | 0.016 | 2.523 |
| 200920_s_at | AL535380 | BTG1 | B-cell translocation gene 1, anti-proliferative | 0.016 | 2.283 |
| 201236_s_at | NM_006763 | BTG2 | BTG family, member 2 | 0.016 | 2.077 |
| 203158_s_at | AF097493 | GLS | glutaminase | 0.016 | 0.420 |
| 204223_at | NM_002725 | PRELP | proline/arginine-rich end leucine-rich repeat protein | 0.016 | 3.935 |
| 204995_at | AL567411 | CDK5R1 | cyclin-dependent kinase 5, regulatory subunit 1 (p35) | 0.016 | 3.989 |
| 211959_at | AW007532 | IGFBP5 | insulin-like growth factor binding protein 5 | 0.016 | 2.714 |
| 212226_s_at | AA628586 | PPAP2B | phosphatidic acid phosphatase type 2B | 0.016 | 3.256 |
| 213496_at | AW592563 | LPPR4 | plasticity related gene 1 | 0.016 | 17.965 |
| 218189_s_at | NM_018946 | NANS | N-acetylneuraminic acid synthase | 0.016 | 2.054 |
| 219247_s_at | NM_024630 | ZDHHC14 | zinc finger, DHHC-type containing 14 | 0.016 | 2.105 |
| 228665_at | AI458003 | CYYR1 | cysteine/tyrosine-rich 1 | 0.016 | 2.847 |
| 202974_at | NM_002436 | MPP1 | membrane protein, palmitoylated 1, 55kDa | 0.016 | 2.351 |
| 203823_at | NM_021106 | RGS3 | regulator of G-protein signaling 3 | 0.016 | 2.376 |
| 206389_s_at | NM_000921 | PDE3A | phosphodiesterase 3A, cGMP-inhibited | 0.016 | 0.345 |
| 213093_at | AI471375 | PRKCA | protein kinase C, alpha | 0.016 | 6.684 |
| 226390_at | AA628398 | STARD4 | StAR-related lipid transfer (START) domain containing 4 | 0.016 | 0.361 |
| 228224_at | AA573140 | PRELP | proline/arginine-rich end leucine-rich repeat protein | 0.016 | 4.063 |
| 37022_at | U41344 | PRELP | proline/arginine-rich end leucine-rich repeat protein | 0.016 | 2.882 |
| 201939_at | NM_006622 | PLK2 | polo-like kinase 2 (Drosophila) | 0.016 | 4.477 |
| 202112_at | NM_000552 | VWF | von Willebrand factor | 0.016 | 4.449 |
| 202340_x_at | NM_002135 | NR4A1 | nuclear receptor subfamily 4, group A, member 1 | 0.016 | 3.899 |
| 203016_s_at | AK001710 | SSX2IP | synovial sarcoma, X breakpoint 2 interacting protein | 0.016 | 0.438 |
| 203017_s_at | R52678 | SSX2IP | synovial sarcoma, X breakpoint 2 interacting protein | 0.016 | 0.451 |
| 203231_s_at | AW235612 | ATXN1 | ataxin 1 | 0.016 | 0.327 |
| 203661_s_at | BC002660 | TMOD1 | tropomodulin 1 | 0.016 | 2.463 |
| 209355_s_at | AB000889 | PPAP2B | phosphatidic acid phosphatase type 2B | 0.016 | 3.456 |
| 212230_at | AV725664 | PPAP2B | phosphatidic acid phosphatase type 2B | 0.016 | 3.379 |
| 212344_at | AW043713 | SULF1 | sulfatase 1 | 0.016 | 5.301 |
| 219025_at | NM_020404 | CD248 | CD248 molecule, endosialin | 0.016 | 4.263 |
| 219197_s_at | AI424243 | SCUBE2 | signal peptide, CUB domain, EGF-like 2 | 0.016 | 3.513 |
| 225263_at | BC001196 | HS6ST1 | heparan sulfate 6-O-sulfotransferase 1 | 0.016 | 3.102 |
| 227915_at | AI872284 | ASB2 | ankyrin repeat and SOCS box-containing 2 | 0.016 | 5.124 |
| 228340_at | BE967118 | TLE3 | transducin-like enhancer of split 3 (E(sp1) homolog, Drosophila) | 0.016 | 2.819 |
| 228771_at | AI651212 | ADRBK2 | adrenergic, beta, receptor kinase 2 | 0.016 | 2.989 |
| 230588_s_at | AA906142 | LOC285074 | hypothetical protein LOC285074 | 0.016 | 5.292 |
| 231773_at | BF002046 | ANGPTL1 | angiopoietin-like 1 | 0.016 | 12.032 |
| 236783_at | AI732844 | KCNIP4 | Kv channel interacting protein 4 | 0.016 | 0.131 |
| 200878_at | AF052094 | EPAS1 | endothelial PAS domain protein 1 | 0.016 | 2.703 |
| 203895_at | AL535113 | PLCB4 | phospholipase C, beta 4 | 0.016 | 0.202 |
| 212345_s_at | BE675139 | CREB3L2 | cAMP responsive element binding protein 3-like 2 | 0.016 | 2.216 |
| 212770_at | AW873621 | TLE3 | transducin-like enhancer of split 3 (E(sp1) homolog, Drosophila) | 0.016 | 2.465 |
| 221039_s_at | NM_018482 | DDEF1 | development and differentiation enhancing factor 1 | 0.016 | 2.058 |
| 222165_x_at | AK022885 | C9orf16 | chromosome 9 open reading frame 16 | 0.016 | 2.339 |
| 225129_at | AW170571 | CPNE2 | copine II | 0.016 | 4.204 |
| 225171_at | BE644830 | ARHGAP18 | Rho GTPase activating protein 18 | 0.016 | 2.715 |
| 228399_at | AI569974 | OSR1 | odd-skipped related 1 (Drosophila) | 0.016 | 0.086 |
| 230250_at | AI670852 | PTPRB | protein tyrosine phosphatase, receptor type, B | 0.016 | 3.069 |
| 230559_x_at | AI277617 | FGD4 | FYVE, RhoGEF and PH domain containing 4 | 0.016 | 0.134 |
| 203018_s_at | AU152583 | SSX2IP | synovial sarcoma, X breakpoint 2 interacting protein | 0.016 | 0.448 |
| 205902_at | AJ251016 | KCNN3 | potassium intermediate/small conductance calcium-activated channel, subfamily N, member 3 | 0.016 | 3.965 |
| 205903_s_at | NM_002249 | KCNN3 | potassium intermediate/small conductance calcium-activated channel, subfamily N, member 3 | 0.016 | 3.967 |
| 206501_x_at | NM_004956 | ETV1 | ets variant gene 1 | 0.016 | 0.434 |
| 209234_at | BF939474 | KIF1B | kinesin family member 1B | 0.016 | 0.451 |
| 212338_at | AA621962 | MYO1D | myosin ID | 0.016 | 2.371 |
| 212353_at | AI479175 | SULF1 | sulfatase 1 | 0.016 | 5.269 |
| 214180_at | AW340588 | MAN1C1 | mannosidase, alpha, class 1C, member 1 | 0.016 | 3.272 |
| 216915_s_at | S69182 | PTPN12 | protein tyrosine phosphatase, non-receptor type 12 | 0.016 | 0.443 |
| 217053_x_at | X87175 | ETV1 | ets variant gene 1 | 0.016 | 0.399 |
| 219557_s_at | NM_020645 | NRIP3 | nuclear receptor interacting protein 3 | 0.016 | 3.642 |
| 223382_s_at | AL136903 | ZNRF1 | zinc and ring finger 1 | 0.016 | 2.182 |
| 223634_at | AF279143 | RASD2 | RASD family, member 2 | 0.016 | 5.187 |
| 224506_s_at | BC006362 | PPAPDC3 | phosphatidic acid phosphatase type 2 domain containing 3 | 0.016 | 2.629 |
| 224530_s_at | AY029176 | KCNIP4 | Kv channel interacting protein 4 | 0.016 | 0.126 |
| 225173_at | BE501862 | ARHGAP18 | Rho GTPase activating protein 18 | 0.016 | 2.896 |
| 228444_at | BF446943 |  |  | 0.016 | 0.459 |
| 229893_at | BF589413 | FRMD3 | FERM domain containing 3 | 0.016 | 4.083 |
| 230645_at | BF110588 | FRMD3 | FERM domain containing 3 | 0.016 | 4.124 |
| 239657_x_at | AI341823 | FOXO6 | forkhead box protein O6 | 0.016 | 0.143 |
| 203019_x_at | NM_014021 | SSX2IP | synovial sarcoma, X breakpoint 2 interacting protein | 0.016 | 0.448 |
| 203896_s_at | NM_000933 | PLCB4 | phospholipase C, beta 4 | 0.016 | 0.201 |
| 205893_at | NM_014932 | NLGN1 | neuroligin 1 | 0.016 | 0.116 |
| 217061_s_at | AC004857 | ETV1 | ets variant gene 1 | 0.016 | 0.415 |
| 222834_s_at | N32508 | GNG12 | guanine nucleotide binding protein (G protein), gamma 12 | 0.016 | 0.424 |
| 225384_at | BF001267 | DOCK7 | dedicator of cytokinesis 7 | 0.016 | 0.465 |
| 227948_at | AI949549 | FGD4 | FYVE, RhoGEF and PH domain containing 4 | 0.016 | 0.186 |
| 229317_at | BG231980 | KPNA5 | karyopherin alpha 5 (importin alpha 6) | 0.016 | 0.477 |
| 231361_at | AI912122 | NLGN1 | neuroligin 1 | 0.016 | 0.118 |
| 243931_at | R64696 |  |  | 0.016 | 0.216 |
| 203662_s_at | NM_003275 | TMOD1 | tropomodulin 1 | 0.016 | 2.341 |
| 215305_at | H79306 | PDGFRA | platelet-derived growth factor receptor, alpha polypeptide | 0.016 | 3.227 |
| 218856_at | NM_016629 | TNFRSF21 | tumor necrosis factor receptor superfamily, member 21 | 0.016 | 2.669 |
| 222451_s_at | BC003128 | ZDHHC9 | zinc finger, DHHC-type containing 9 | 0.016 | 2.082 |
| 226028_at | AA156022 | ROBO4 | roundabout homolog 4, magic roundabout (Drosophila) | 0.016 | 3.544 |
| 234725_s_at | AK026133 | SEMA4B | sema domain, immunoglobulin domain (Ig), transmembrane domain (TM) and short cytoplasmic domain, (semaphorin) 4B | 0.016 | 2.242 |
| 235019_at | BE878495 | CPM | carboxypeptidase M | 0.016 | 3.176 |
| 235706_at | AW663908 | CPM | carboxypeptidase M | 0.016 | 3.144 |
| 239118_at | BF513715 | KCNA2 | potassium voltage-gated channel, shaker-related subfamily, member 2 | 0.016 | 4.147 |
| 201369_s_at | NM_006887 | ZFP36L2 | zinc finger protein 36, C3H type-like 2 | 0.017 | 2.295 |
| 201681_s_at | AB011155 | DLG5 | discs, large homolog 5 (Drosophila) | 0.017 | 2.141 |
| 202796_at | NM_007286 | SYNPO | synaptopodin | 0.017 | 3.786 |
| 203233_at | NM_000418 | IL4R | interleukin 4 receptor | 0.017 | 3.705 |
| 203723_at | NM_002221 | ITPKB | inositol 1,4,5-trisphosphate 3-kinase B | 0.017 | 3.032 |
| 205111_s_at | NM_016341 | PLCE1 | phospholipase C, epsilon 1 | 0.017 | 0.353 |
| 205112_at | NM_016341 | PLCE1 | phospholipase C, epsilon 1 | 0.017 | 0.342 |
| 210839_s_at | D45421 | ENPP2 | ectonucleotide pyrophosphatase/phosphodiesterase 2 | 0.017 | 2.447 |
| 221529_s_at | AF326591 | PLVAP | plasmalemma vesicle associated protein | 0.017 | 3.535 |
| 223877_at | AF329839 | C1QTNF7 | C1q and tumor necrosis factor related protein 7 | 0.017 | 0.172 |
| 227307_at | AL565381 | TSPAN18 | tetraspanin 18 | 0.017 | 4.178 |
| 228108_at | AW274846 |  |  | 0.017 | 3.324 |
| 228776_at | AA430014 | GJC1 | gap junction protein, gamma 1, 45kDa | 0.017 | 5.175 |
| 236300_at | BF698797 |  |  | 0.017 | 0.496 |
| 241765_at | AI469884 | CPM | carboxypeptidase M | 0.017 | 2.904 |
| 1569433_at | BC020896 | SAMD5 | sterile alpha motif domain containing 5 | 0.017 | 0.247 |
| 203131_at | NM_006206 | PDGFRA | platelet-derived growth factor receptor, alpha polypeptide | 0.017 | 2.054 |
| 206241_at | NM_002269 | KPNA5 | karyopherin alpha 5 (importin alpha 6) | 0.017 | 0.380 |
| 210871_x_at | AL133046 | SSX2IP | synovial sarcoma, X breakpoint 2 interacting protein | 0.017 | 0.443 |
| 212314_at | AB018289 | KIAA0746 | KIAA0746 protein | 0.017 | 6.096 |
| 212354_at | BE500977 | SULF1 | sulfatase 1 | 0.017 | 5.354 |
| 216997_x_at | AL358975 | TLE4 | transducin-like enhancer of split 4 (E(sp1) homolog, Drosophila) | 0.017 | 0.288 |
| 217821_s_at | AF118023 | WBP11 | WW domain binding protein 11 | 0.017 | 2.039 |
| 218918_at | NM_020379 | MAN1C1 | mannosidase, alpha, class 1C, member 1 | 0.017 | 3.524 |
| 222108_at | AC004010 | AMIGO2 | adhesion molecule with Ig-like domain 2 | 0.017 | 0.166 |
| 224339_s_at | AB056476 | ANGPTL1 | angiopoietin-like 1 | 0.017 | 12.677 |
| 225166_at | AU158022 | ARHGAP18 | Rho GTPase activating protein 18 | 0.017 | 2.760 |
| 225664_at | AA788946 | COL12A1 | collagen, type XII, alpha 1 | 0.017 | 4.979 |
| 235044_at | H06649 | CYYR1 | cysteine/tyrosine-rich 1 | 0.017 | 2.999 |
| 240015_at | AI299467 |  |  | 0.017 | 0.342 |
| 243403_x_at | R28370 | CPM | carboxypeptidase M | 0.017 | 2.975 |
| 244040_at | N47474 |  |  | 0.017 | 2.929 |
| 1555240_s_at | AF493879 | GNG12 | guanine nucleotide binding protein (G protein), gamma 12 | 0.017 | 0.381 |
| 201645_at | NM_002160 | TNC | tenascin C | 0.017 | 4.454 |
| 202883_s_at | T79584 | PPP2R1B | protein phosphatase 2 (formerly 2A), regulatory subunit A, beta isoform | 0.017 | 3.148 |
| 203837_at | NM_005923 | MAP3K5 | mitogen-activated protein kinase kinase kinase 5 | 0.017 | 2.070 |
| 210381_s_at | BC000740 | CCKBR | cholecystokinin B receptor | 0.017 | 0.204 |
| 211685_s_at | AF251061 | NCALD | neurocalcin delta | 0.017 | 2.593 |
| 212311_at | AA522514 | KIAA0746 | KIAA0746 protein | 0.017 | 6.991 |
| 214581_x_at | BE568134 | TNFRSF21 | tumor necrosis factor receptor superfamily, member 21 | 0.017 | 2.662 |
| 217057_s_at | AF107846 | GNAS | GNAS complex locus | 0.017 | 10.897 |
| 222912_at | BE207758 | ARRB1 | arrestin, beta 1 | 0.017 | 0.484 |
| 223595_at | AF247167 | TMEM133 | transmembrane protein 133 | 0.017 | 3.374 |
| 225383_at | BF793625 | ZNF275 | zinc finger protein 275 | 0.017 | 2.249 |
| 228055_at | AI763426 | NAPSB | napsin B aspartic peptidase pseudogene | 0.017 | 3.842 |
| 228457_at | AI590190 |  |  | 0.017 | 2.777 |
| 228977_at | AI669535 | LOC729680 | hypothetical protein LOC729680 | 0.017 | 0.035 |
| 229506_at | BF114646 |  |  | 0.017 | 2.687 |
| 231973_s_at | AK001223 | ANAPC1 | anaphase promoting complex subunit 1 | 0.017 | 3.172 |
| 239349_at | BE856929 | C1QTNF7 | C1q and tumor necrosis factor related protein 7 | 0.017 | 0.216 |
| 202084_s_at | NM_003003 | SEC14L1 | SEC14-like 1 (S. cerevisiae) | 0.019 | 2.146 |
| 203147_s_at | BE962483 | TRIM14 | tripartite motif-containing 14 | 0.019 | 2.656 |
| 203148_s_at | NM_014788 | TRIM14 | tripartite motif-containing 14 | 0.019 | 2.309 |
| 203632_s_at | NM_016235 | GPRC5B | G protein-coupled receptor, family C, group 5, member B | 0.019 | 2.683 |
| 203836_s_at | D84476 | MAP3K5 | mitogen-activated protein kinase kinase kinase 5 | 0.019 | 2.532 |
| 205712_at | NM_002839 | PTPRD | protein tyrosine phosphatase, receptor type, D | 0.019 | 0.134 |
| 206100_at | NM_001874 | CPM | carboxypeptidase M | 0.019 | 2.896 |
| 208962_s_at | BE540552 | FADS1 | fatty acid desaturase 1 | 0.019 | 0.280 |
| 209146_at | AV704962 | SC4MOL | sterol-C4-methyl oxidase-like | 0.019 | 0.489 |
| 213388_at | H15535 | PDE4DIP | phosphodiesterase 4D interacting protein | 0.019 | 0.427 |
| 214767_s_at | AL551046 | HSPB6 | heat shock protein, alpha-crystallin-related, B6 | 0.019 | 2.756 |
| 221858_at | N34407 | TBC1D12 | TBC1 domain family, member 12 | 0.019 | 0.309 |
| 223079_s_at | AI828035 | GLS | glutaminase | 0.019 | 0.404 |
| 224583_at | AL565621 | COTL1 | coactosin-like 1 (Dictyostelium) | 0.019 | 2.321 |
| 224932_at | AI814909 | CHCHD10 | coiled-coil-helix-coiled-coil-helix domain containing 10 | 0.019 | 2.306 |
| 225382_at | U82670 | ZNF275 | zinc finger protein 275 | 0.019 | 2.205 |
| 227417_at | AW057543 | MOSC2 | MOCO sulphurase C-terminal domain containing 2 | 0.019 | 0.199 |
| 227550_at | AW242720 | LOC143381 | hypothetical protein LOC143381 | 0.019 | 2.717 |
| 227657_at | AA722069 | RNF150 | ring finger protein 150 | 0.019 | 0.212 |
| 238865_at | AI822134 | PABPC4L | poly(A) binding protein, cytoplasmic 4-like | 0.019 | 0.497 |
| 244647_at | AA233885 |  |  | 0.019 | 2.189 |
| 1555997_s_at | BM128432 | IGFBP5 | insulin-like growth factor binding protein 5 | 0.020 | 3.398 |
| 201625_s_at | BE300521 | INSIG1 | insulin induced gene 1 | 0.020 | 0.343 |
| 202995_s_at | NM_006486 | FBLN1 | fibulin 1 | 0.020 | 2.923 |
| 203425_s_at | NM_000599 | IGFBP5 | insulin-like growth factor binding protein 5 | 0.020 | 3.253 |
| 203426_s_at | M65062 | IGFBP5 | insulin-like growth factor binding protein 5 | 0.020 | 3.308 |
| 204803_s_at | NM_004165 | RRAD | Ras-related associated with diabetes | 0.020 | 0.380 |
| 208963_x_at | BG165833 | FADS1 | fatty acid desaturase 1 | 0.020 | 0.338 |
| 209365_s_at | U65932 | ECM1 | extracellular matrix protein 1 | 0.020 | 3.936 |
| 210831_s_at | L27489 | PTGER3 | prostaglandin E receptor 3 (subtype EP3) | 0.020 | 0.197 |
| 212646_at | D42043 | RFTN1 | raftlin, lipid raft linker 1 | 0.020 | 4.236 |
| 213792_s_at | AA485908 | INSR | insulin receptor | 0.020 | 2.052 |
| 222351_at | AW009884 | PPP2R1B | protein phosphatase 2 (formerly 2A), regulatory subunit A, beta isoform | 0.020 | 13.243 |
| 222900_at | AJ400877 |  |  | 0.020 | 4.011 |
| 225867_at | BE741869 | VASN | vasorin | 0.020 | 2.540 |
| 235527_at | U55983 | LOC284214 | hypothetical protein LOC284214 | 0.020 | 0.136 |
| 236038_at | N50714 |  |  | 0.020 | 0.293 |
| 202948_at | NM_000877 | IL1R1 | interleukin 1 receptor, type I | 0.021 | 3.032 |
| 203424_s_at | AW157548 | IGFBP5 | insulin-like growth factor binding protein 5 | 0.021 | 3.986 |
| 203934_at | NM_002253 | KDR | kinase insert domain receptor (a type III receptor tyrosine kinase) | 0.021 | 2.820 |
| 204595_s_at | AI300520 | STC1 | stanniocalcin 1 | 0.021 | 3.537 |
| 204639_at | NM_000022 | ADA | adenosine deaminase | 0.021 | 2.557 |
| 205501_at | AI143879 | PDE10A | phosphodiesterase 10A | 0.021 | 3.332 |
| 206187_at | NM_000960 | PTGIR | prostaglandin I2 (prostacyclin) receptor (IP) | 0.021 | 2.775 |
| 212256_at | BE906572 | GALNT10 | UDP-N-acetyl-alpha-D-galactosamine:polypeptide N-acetylgalactosaminyltransferase 10 (GalNAc-T10) | 0.021 | 2.200 |
| 213013_at | BG164295 | MAPK8IP1 | mitogen-activated protein kinase 8 interacting protein 1 | 0.021 | 2.708 |
| 213100_at | AA127885 |  |  | 0.021 | 2.440 |
| 216598_s_at | S69738 | CCL2 | chemokine (C-C motif) ligand 2 | 0.021 | 2.845 |
| 216705_s_at | X02189 | ADA | adenosine deaminase | 0.021 | 2.600 |
| 219155_at | NM_012417 | PITPNC1 | phosphatidylinositol transfer protein, cytoplasmic 1 | 0.021 | 2.040 |
| 222653_at | AA005137 | PNPO | pyridoxamine 5'-phosphate oxidase | 0.021 | 2.002 |
| 223383_at | AL136903 | ZNRF1 | zinc and ring finger 1 | 0.021 | 2.174 |
| 226899_at | AK022859 | UNC5B | unc-5 homolog B (C. elegans) | 0.021 | 2.482 |
| 235301_at | AI797353 | KIAA1324L | KIAA1324-like | 0.021 | 2.404 |
| 205227_at | NM_002182 | IL1RAP | interleukin 1 receptor accessory protein | 0.021 | 0.301 |
| 207992_s_at | NM_000480 | AMPD3 | adenosine monophosphate deaminase (isoform E) | 0.021 | 0.369 |
| 208964_s_at | AL512760 | FADS1 | fatty acid desaturase 1 | 0.021 | 0.276 |
| 209491_s_at | AA919119 | AMPD3 | adenosine monophosphate deaminase (isoform E) | 0.021 | 0.271 |
| 210374_x_at | D38300 | PTGER3 | prostaglandin E receptor 3 (subtype EP3) | 0.021 | 0.189 |
| 211744_s_at | BC005930 | CD58 | CD58 molecule | 0.021 | 0.311 |
| 216942_s_at | D28586 | CD58 | CD58 molecule | 0.021 | 0.308 |
| 219527_at | NM_017898 | MOSC2 | MOCO sulphurase C-terminal domain containing 2 | 0.021 | 0.260 |
| 222256_s_at | AK000550 | JMJD7 | jumonji domain containing 7 | 0.021 | 0.487 |
| 227415_at | BF109303 | DGKH | diacylglycerol kinase, eta | 0.021 | 0.286 |
| 240890_at | AA041298 | LOC643733 | hypothetical LOC643733 | 0.021 | 0.406 |
| 1555106_a_at | BC035744 | CTDSPL2 | CTD (carboxy-terminal domain, RNA polymerase II, polypeptide A) small phosphatase like 2 | 0.022 | 0.417 |
| 202884_s_at | NM_002716 | PPP2R1B | protein phosphatase 2 (formerly 2A), regulatory subunit A, beta isoform | 0.022 | 5.421 |
| 203232_s_at | NM_000332 | ATXN1 | ataxin 1 | 0.022 | 0.482 |
| 203711_s_at | NM_014362 | HIBCH | 3-hydroxyisobutyryl-Coenzyme A hydrolase | 0.022 | 0.452 |
| 209197_at | AA626780 | SYT11 | synaptotagmin XI | 0.022 | 0.493 |
| 209543_s_at | M81104 | CD34 | CD34 molecule | 0.022 | 0.359 |
| 221636_s_at | AL136931 | MOSC2 | MOCO sulphurase C-terminal domain containing 2 | 0.022 | 0.279 |
| 224061_at | AF128846 | INMT | indolethylamine N-methyltransferase | 0.022 | 0.171 |
| 225033_at | AV721528 | LOC286167 | hypothetical LOC286167 | 0.022 | 3.171 |
| 226304_at | AA563621 | HSPB6 | heat shock protein, alpha-crystallin-related, B6 | 0.022 | 3.006 |
| 228438_at | AI948599 | LOC100132891 | hypothetical protein LOC100132891 | 0.022 | 0.286 |
| 229024_at | BF056892 |  |  | 0.022 | 0.293 |
| 230746_s_at | AW003173 | STC1 | stanniocalcin 1 | 0.022 | 3.463 |
| 233116_at | U82695 |  |  | 0.022 | 3.557 |
| 235252_at | AI090141 | KSR1 | kinase suppressor of ras 1 | 0.022 | 2.031 |
| 201791_s_at | NM_001360 | DHCR7 | 7-dehydrocholesterol reductase | 0.024 | 0.459 |
| 203910_at | NM_004815 | ARHGAP29 | Rho GTPase activating protein 29 | 0.024 | 2.818 |
| 204368_at | NM_005630 | SLCO2A1 | solute carrier organic anion transporter family, member 2A1 | 0.024 | 3.569 |
| 205173_x_at | NM_001779 | CD58 | CD58 molecule | 0.024 | 0.356 |
| 205429_s_at | NM_016447 | MPP6 | membrane protein, palmitoylated 6 (MAGUK p55 subfamily member 6) | 0.024 | 0.409 |
| 206472_s_at | NM_005078 | TLE3 | transducin-like enhancer of split 3 (E(sp1) homolog, Drosophila) | 0.024 | 2.214 |
| 206510_at | AF332197 | SIX2 | SIX homeobox 2 | 0.024 | 3.340 |
| 210198_s_at | BC002665 | PLP1 | proteolipid protein 1 | 0.024 | 32.131 |
| 212472_at | BE965029 | MICAL2 | microtubule associated monoxygenase, calponin and LIM domain containing 2 | 0.024 | 4.184 |
| 212724_at | BG054844 | RND3 | Rho family GTPase 3 | 0.024 | 0.246 |
| 218832_x_at | NM_004041 | ARRB1 | arrestin, beta 1 | 0.024 | 0.450 |
| 226756_at | AA191741 |  |  | 0.024 | 2.119 |
| 227420_at | BF338045 | TNFAIP8L1 | tumor necrosis factor, alpha-induced protein 8-like 1 | 0.024 | 2.687 |
| 230006_s_at | AI742358 | SVIP | small VCP/p97-interacting protein | 0.024 | 0.481 |
| 230163_at | AW263087 | LOC143381 | hypothetical protein LOC143381 | 0.024 | 2.466 |
| 1554029_a_at | BC030966 | TTC37 | tetratricopeptide repeat domain 37 | 0.025 | 0.432 |
| 202242_at | NM_004615 | TSPAN7 | tetraspanin 7 | 0.025 | 3.007 |
| 202524_s_at | NM_014767 | SPOCK2 | sparc/osteonectin, cwcv and kazal-like domains proteoglycan (testican) 2 | 0.025 | 6.232 |
| 202994_s_at | Z95331 | FBLN1 | fibulin 1 | 0.025 | 2.602 |
| 203130_s_at | NM_004522 | KIF5C | kinesin family member 5C | 0.025 | 0.167 |
| 204677_at | NM_001795 | CDH5 | cadherin 5, type 2 (vascular endothelium) | 0.025 | 2.991 |
| 209051_s_at | AF295773 | RALGDS | ral guanine nucleotide dissociation stimulator | 0.025 | 2.167 |
| 209064_x_at | AL136920 | PAIP1 | poly(A) binding protein interacting protein 1 | 0.025 | 0.491 |
| 209436_at | AB018305 | SPON1 | spondin 1, extracellular matrix protein | 0.025 | 3.268 |
| 209540_at | AU144912 | IGF1 | insulin-like growth factor 1 (somatomedin C) | 0.025 | 5.764 |
| 210512_s_at | AF022375 | VEGFA | vascular endothelial growth factor A | 0.025 | 3.088 |
| 210738_s_at | AF011390 | SLC4A4 | solute carrier family 4, sodium bicarbonate cotransporter, member 4 | 0.025 | 0.080 |
| 210809_s_at | D13665 | POSTN | periostin, osteoblast specific factor | 0.025 | 0.086 |
| 212473_s_at | BE965029 | MICAL2 | microtubule associated monoxygenase, calponin and LIM domain containing 2 | 0.025 | 3.800 |
| 213001_at | AF007150 | ANGPTL2 | angiopoietin-like 2 | 0.025 | 3.363 |
| 213358_at | AB018345 | KIAA0802 | KIAA0802 | 0.025 | 3.281 |
| 215022_x_at | BG429214 | ZNF33B | zinc finger protein 33B | 0.025 | 0.499 |
| 218223_s_at | NM_016274 | PLEKHO1 | pleckstrin homology domain containing, family O member 1 | 0.025 | 2.061 |
| 220006_at | NM_024768 | CCDC48 | coiled-coil domain containing 48 | 0.025 | 3.207 |
| 222154_s_at | AK002064 | LOC26010 | viral DNA polymerase-transactivated protein 6 | 0.025 | 0.414 |
| 225536_at | AL545105 | TMEM54 | transmembrane protein 54 | 0.025 | 0.400 |
| 225606_at | AI949179 | BCL2L11 | BCL2-like 11 (apoptosis facilitator) | 0.025 | 2.435 |
| 226582_at | AL520272 | LOC400043 | hypothetical gene supported by BC009385 | 0.025 | 3.374 |
| 239598_s_at | AA789296 | LPCAT2 | lysophosphatidylcholine acyltransferase 2 | 0.025 | 0.178 |
| 1554239_s_at | BC033780 | ZADH2 | zinc binding alcohol dehydrogenase domain containing 2 | 0.027 | 0.451 |
| 201539_s_at | U29538 | FHL1 | four and a half LIM domains 1 | 0.027 | 0.469 |
| 201655_s_at | M85289 | HSPG2 | heparan sulfate proteoglycan 2 | 0.027 | 3.020 |
| 205808_at | NM_004318 | ASPH | aspartate beta-hydroxylase | 0.027 | 0.462 |
| 208051_s_at | NM_006451 | PAIP1 | poly(A) binding protein interacting protein 1 | 0.027 | 0.482 |
| 209437_s_at | AB051390 | SPON1 | spondin 1, extracellular matrix protein | 0.027 | 3.606 |
| 210832_x_at | D38298 | PTGER3 | prostaglandin E receptor 3 (subtype EP3) | 0.027 | 0.187 |
| 212843_at | AA126505 | NCAM1 | neural cell adhesion molecule 1 | 0.027 | 3.020 |
| 213993_at | AI885290 | SPON1 | spondin 1, extracellular matrix protein | 0.027 | 3.973 |
| 218145_at | NM_021158 | TRIB3 | tribbles homolog 3 (Drosophila) | 0.027 | 0.385 |
| 218683_at | NM_021190 | PTBP2 | polypyrimidine tract binding protein 2 | 0.027 | 0.335 |
| 219232_s_at | NM_022073 | EGLN3 | egl nine homolog 3 (C. elegans) | 0.027 | 3.737 |
| 222033_s_at | AA058828 | FLT1 | fms-related tyrosine kinase 1 (vascular endothelial growth factor/vascular permeability factor receptor) | 0.027 | 2.670 |
| 226497_s_at | AA149648 |  |  | 0.027 | 2.626 |
| 226782_at | BF001919 | SLC25A30 | solute carrier family 25, member 30 | 0.027 | 0.338 |
| 228653_at | AI700341 | SAMD5 | sterile alpha motif domain containing 5 | 0.027 | 0.379 |
| 230715_at | AI138969 | ZNF518B | zinc finger protein 518B | 0.027 | 0.412 |
| 238480_at | AI871745 |  |  | 0.027 | 2.931 |
| 40016_g_at | AB002301 | MAST4 | microtubule associated serine/threonine kinase family member 4 | 0.027 | 2.135 |
| 1555778_a_at | AY140646 | POSTN | periostin, osteoblast specific factor | 0.029 | 0.062 |
| 200974_at | NM_001613 | ACTA2 | actin, alpha 2, smooth muscle, aorta | 0.029 | 2.982 |
| 203780_at | AF275945 | MPZL2 | myelin protein zero-like 2 | 0.029 | 2.707 |
| 203888_at | NM_000361 | THBD | thrombomodulin | 0.029 | 6.317 |
| 205680_at | NM_002425 | MMP10 | matrix metallopeptidase 10 (stromelysin 2) | 0.029 | 2.434 |
| 209281_s_at | M95541 | ATP2B1 | ATPase, Ca++ transporting, plasma membrane 1 | 0.029 | 2.158 |
| 209541_at | AI972496 | IGF1 | insulin-like growth factor 1 (somatomedin C) | 0.029 | 5.139 |
| 210833_at | AL031429 | PTGER3 | prostaglandin E receptor 3 (subtype EP3) | 0.029 | 0.144 |
| 219858_s_at | NM_017694 | FLJ20160 | FLJ20160 protein | 0.029 | 0.470 |
| 222759_at | BC002522 | SUV420H1 | suppressor of variegation 4-20 homolog 1 (Drosophila) | 0.029 | 0.499 |
| 222833_at | AU154202 | LPCAT2 | lysophosphatidylcholine acyltransferase 2 | 0.029 | 0.253 |
| 223130_s_at | AF212221 | MYLIP | myosin regulatory light chain interacting protein | 0.029 | 0.309 |
| 230087_at | AI823645 | PRIMA1 | proline rich membrane anchor 1 | 0.029 | 6.360 |
| 230109_at | AI638433 | PDE7B | phosphodiesterase 7B | 0.029 | 4.524 |
| 230849_at | N64750 | KCNA1 | potassium voltage-gated channel, shaker-related subfamily, member 1 (episodic ataxia with myokymia) | 0.029 | 2.939 |
| 233868_x_at | AL117415 | ADAM33 | ADAM metallopeptidase domain 33 | 0.029 | 2.119 |
| 235952_at | AA521504 |  |  | 0.029 | 0.367 |
| 240145_at | AW628059 |  |  | 0.029 | 0.358 |
| 241399_at | AI142028 | FAM19A2 | family with sequence similarity 19 (chemokine (C-C motif)-like), member A2 | 0.029 | 0.066 |
| 202897_at | AB023430 | SIRPA | signal-regulatory protein alpha | 0.030 | 2.710 |
| 203509_at | NM_003105 | SORL1 | sortilin-related receptor, L(DLR class) A repeats-containing | 0.030 | 2.927 |
| 203903_s_at | NM_014799 | HEPH | hephaestin | 0.030 | 2.124 |
| 203904_x_at | NM_002231 | CD82 | CD82 molecule | 0.030 | 2.757 |
| 205709_s_at | NM_001263 | CDS1 | CDP-diacylglycerol synthase (phosphatidate cytidylyltransferase) 1 | 0.030 | 0.271 |
| 206201_s_at | NM_005924 | MEOX2 | mesenchyme homeobox 2 | 0.030 | 4.671 |
| 206511_s_at | NM_016932 | SIX2 | SIX homeobox 2 | 0.030 | 3.128 |
| 206540_at | NM_024506 | GLB1L | galactosidase, beta 1-like | 0.030 | 3.768 |
| 208102_s_at | NM_002779 | PSD | pleckstrin and Sec7 domain containing | 0.030 | 2.596 |
| 209589_s_at | AF025304 | EPHB2 | EPH receptor B2 | 0.030 | 2.871 |
| 213562_s_at | BF979497 | SQLE | squalene epoxidase | 0.030 | 0.468 |
| 213994_s_at | AI885290 | SPON1 | spondin 1, extracellular matrix protein | 0.030 | 2.864 |
| 214319_at | W58342 | FRY | furry homolog (Drosophila) | 0.030 | 0.260 |
| 215271_at | BF432086 | TNN | tenascin N | 0.030 | 3.556 |
| 219514_at | NM_012098 | ANGPTL2 | angiopoietin-like 2 | 0.030 | 3.216 |
| 224836_at | AL109824 | TP53INP2 | tumor protein p53 inducible nuclear protein 2 | 0.030 | 2.042 |
| 227318_at | AL359605 |  |  | 0.030 | 4.577 |
| 227526_at | AU151222 | CDON | Cdon homolog (mouse) | 0.030 | 3.194 |
| 229175_at | AI971520 | SMYD4 | SET and MYND domain containing 4 | 0.030 | 0.408 |
| 235944_at | BF446673 | HMCN1 | hemicentin 1 | 0.030 | 0.426 |
| 242326_at | AA977081 | COL22A1 | collagen, type XXII, alpha 1 | 0.030 | 5.184 |
| 1561180_at | AK021807 |  |  | 0.032 | 2.466 |
| 203887_s_at | NM_000361 | THBD | thrombomodulin | 0.032 | 7.051 |
| 204043_at | NM_000355 | TCN2 | transcobalamin II; macrocytic anemia | 0.032 | 2.378 |
| 204396_s_at | NM_005308 | GRK5 | G protein-coupled receptor kinase 5 | 0.032 | 2.275 |
| 205651_x_at | NM_007023 | RAPGEF4 | Rap guanine nucleotide exchange factor (GEF) 4 | 0.032 | 4.779 |
| 205987_at | NM_001765 | CD1C | CD1c molecule | 0.032 | 2.810 |
| 206682_at | NM_006344 | CLEC10A | C-type lectin domain family 10, member A | 0.032 | 2.619 |
| 206932_at | NM_003956 | CH25H | cholesterol 25-hydroxylase | 0.032 | 7.993 |
| 208502_s_at | NM_002653 | PITX1 | paired-like homeodomain 1 | 0.032 | 0.150 |
| 208981_at | AA702701 | PECAM1 | platelet/endothelial cell adhesion molecule | 0.032 | 2.434 |
| 209894_at | U50748 | LEPR | leptin receptor | 0.032 | 0.449 |
| 212805_at | AB002365 | PRUNE2 | prune homolog 2 (Drosophila) | 0.032 | 3.237 |
| 212906_at | BE044440 | GRAMD1B | GRAM domain containing 1B | 0.032 | 2.077 |
| 218806_s_at | AF118887 | VAV3 | vav 3 guanine nucleotide exchange factor | 0.032 | 2.208 |
| 218807_at | NM_006113 | VAV3 | vav 3 guanine nucleotide exchange factor | 0.032 | 2.027 |
| 219867_at | NM_024944 | CHODL | chondrolectin | 0.032 | 0.228 |
| 225262_at | AI670862 | FOSL2 | FOS-like antigen 2 | 0.032 | 2.072 |
| 225288_at | AI949136 | COL27A1 | collagen, type XXVII, alpha 1 | 0.032 | 2.141 |
| 225337_at | AI346910 | ABHD2 | abhydrolase domain containing 2 | 0.032 | 2.034 |
| 225379_at | AA199717 | MAPT | microtubule-associated protein tau | 0.032 | 3.726 |
| 225970_at | AA029818 | DDHD1 | DDHD domain containing 1 | 0.032 | 0.430 |
| 227032_at | AI694545 | PLXNA2 | plexin A2 | 0.032 | 2.290 |
| 227080_at | AW003092 | ZNF697 | zinc finger protein 697 | 0.032 | 0.390 |
| 227889_at | AI765437 | LPCAT2 | lysophosphatidylcholine acyltransferase 2 | 0.032 | 0.303 |
| 238823_at | AA481044 | FMNL3 | formin-like 3 | 0.032 | 2.108 |
| 244317_at | BF035563 | KIAA1324L | KIAA1324-like | 0.032 | 2.471 |
| 1570552_at | AF363068 | C18orf50 | chromosome 18 open reading frame 50 | 0.034 | 2.238 |
| 204082_at | NM_006195 | PBX3 | pre-B-cell leukemia homeobox 3 | 0.034 | 2.883 |
| 204165_at | NM_003931 | WASF1 | WAS protein family, member 1 | 0.034 | 2.331 |
| 205174_s_at | NM_012413 | QPCT | glutaminyl-peptide cyclotransferase | 0.034 | 4.624 |
| 205696_s_at | NM_005264 | GFRA1 | GDNF family receptor alpha 1 | 0.034 | 2.221 |
| 206757_at | NM_001083 | PDE5A | phosphodiesterase 5A, cGMP-specific | 0.034 | 0.479 |
| 209198_s_at | BC004291 | SYT11 | synaptotagmin XI | 0.034 | 0.469 |
| 210605_s_at | BC003610 | MFGE8 | milk fat globule-EGF factor 8 protein | 0.034 | 4.443 |
| 211555_s_at | AF020340 | GUCY1B3 | guanylate cyclase 1, soluble, beta 3 | 0.034 | 0.485 |
| 212282_at | BF038366 | TMEM97 | transmembrane protein 97 | 0.034 | 3.731 |
| 213362_at | N73931 | PTPRD | protein tyrosine phosphatase, receptor type, D | 0.034 | 0.198 |
| 214043_at | BF062299 | PTPRD | protein tyrosine phosphatase, receptor type, D | 0.034 | 0.185 |
| 214318_s_at | W58342 | FRY | furry homolog (Drosophila) | 0.034 | 0.288 |
| 219440_at | NM_021785 | RAI2 | retinoic acid induced 2 | 0.034 | 2.222 |
| 220319_s_at | NM_013262 | MYLIP | myosin regulatory light chain interacting protein | 0.034 | 0.275 |
| 221899_at | AI809961 | N4BP2L2 | NEDD4 binding protein 2-like 2 | 0.034 | 0.491 |
| 223129_x_at | T63512 | MYLIP | myosin regulatory light chain interacting protein | 0.034 | 0.314 |
| 226433_at | BF056204 | RNF157 | ring finger protein 157 | 0.034 | 2.138 |
| 228485_s_at | AW165999 | SLC44A1 | solute carrier family 44, member 1 | 0.034 | 0.491 |
| 228618_at | AL040178 | PEAR1 | platelet endothelial aggregation receptor 1 | 0.034 | 0.322 |
| 229084_at | R42166 | CNTN4 | contactin 4 | 0.034 | 3.823 |
| 229670_at | BF056369 |  |  | 0.034 | 2.500 |
| 239039_at | AI859031 |  |  | 0.034 | 4.425 |
| 1555269_a_at | BC033036 | ANO1 | anoctamin 1, calcium activated chloride channel | 0.037 | 0.453 |
| 200862_at | NM_014762 | DHCR24 | 24-dehydrocholesterol reductase | 0.037 | 0.278 |
| 203908_at | NM_003759 | SLC4A4 | solute carrier family 4, sodium bicarbonate cotransporter, member 4 | 0.037 | 0.098 |
| 204894_s_at | NM_003734 | AOC3 | amine oxidase, copper containing 3 (vascular adhesion protein 1) | 0.037 | 7.032 |
| 204923_at | AL023653 | SASH3 | SAM and SH3 domain containing 3 | 0.037 | 2.275 |
| 204955_at | NM_006307 | SRPX | sushi-repeat-containing protein, X-linked | 0.037 | 3.191 |
| 205392_s_at | NM_004166 | CCL14 | chemokine (C-C motif) ligand 14 | 0.037 | 6.137 |
| 205952_at | NM_002246 | KCNK3 | potassium channel, subfamily K, member 3 | 0.037 | 2.233 |
| 211165_x_at | D31661 | EPHB2 | EPH receptor B2 | 0.037 | 4.245 |
| 213004_at | AI074333 | ANGPTL2 | angiopoietin-like 2 | 0.037 | 2.975 |
| 214920_at | R33964 | THSD7A | thrombospondin, type I, domain containing 7A | 0.037 | 0.279 |
| 216264_s_at | X79683 | LAMB2 | laminin, beta 2 (laminin S) | 0.037 | 2.119 |
| 217983_s_at | NM_003730 | RNASET2 | ribonuclease T2 | 0.037 | 2.341 |
| 218469_at | NM_013372 | GREM1 | gremlin 1, cysteine knot superfamily, homolog (Xenopus laevis) | 0.037 | 0.080 |
| 218613_at | NM_018422 | PSD3 | pleckstrin and Sec7 domain containing 3 | 0.037 | 2.076 |
| 225061_at | N45231 | DNAJA4 | DnaJ (Hsp40) homolog, subfamily A, member 4 | 0.037 | 2.426 |
| 225464_at | N30138 | FRMD6 | FERM domain containing 6 | 0.037 | 2.094 |
| 225481_at | AL040051 | FRMD6 | FERM domain containing 6 | 0.037 | 2.003 |
| 226498_at | AA149648 |  |  | 0.037 | 2.818 |
| 226818_at | T64884 | MPEG1 | macrophage expressed gene 1 | 0.037 | 2.080 |
| 201578_at | NM_005397 | PODXL | podocalyxin-like | 0.040 | 2.003 |
| 203423_at | NM_002899 | RBP1 | retinol binding protein 1, cellular | 0.040 | 2.010 |
| 204404_at | NM_001046 | SLC12A2 | solute carrier family 12 (sodium/potassium/chloride transporters), member 2 | 0.040 | 0.483 |
| 205080_at | NM_000965 | RARB | retinoic acid receptor, beta | 0.040 | 2.374 |
| 205559_s_at | NM_006200 | PCSK5 | proprotein convertase subtilisin/kexin type 5 | 0.040 | 2.014 |
| 207375_s_at | NM_002189 | IL15RA | interleukin 15 receptor, alpha | 0.040 | 3.348 |
| 208530_s_at | NM_016152 | RARB | retinoic acid receptor, beta | 0.040 | 2.466 |
| 208850_s_at | AL558479 | THY1 | Thy-1 cell surface antigen | 0.040 | 2.018 |
| 208998_at | U94592 | UCP2 | uncoupling protein 2 (mitochondrial, proton carrier) | 0.040 | 2.492 |
| 209236_at | AL389886 | SLC23A2 | solute carrier family 23 (nucleobase transporters), member 2 | 0.040 | 0.351 |
| 213869_x_at | AA218868 | THY1 | Thy-1 cell surface antigen | 0.040 | 2.015 |
| 213933_at | AW242315 | PTGER3 | prostaglandin E receptor 3 (subtype EP3) | 0.040 | 0.242 |
| 214660_at | X68742 | ITGA1 | integrin, alpha 1 | 0.040 | 0.290 |
| 217738_at | BF575514 | NAMPT | nicotinamide phosphoribosyltransferase | 0.040 | 2.846 |
| 218472_s_at | NM_015946 | PELO | pelota homolog (Drosophila) | 0.040 | 0.434 |
| 227325_at | AW172584 | LOC255783 | hypothetical protein LOC255783 | 0.040 | 2.327 |
| 228098_s_at | AW292746 | MYLIP | myosin regulatory light chain interacting protein | 0.040 | 0.342 |
| 235033_at | AL577823 | NPEPL1 | aminopeptidase-like 1 | 0.040 | 2.145 |
| 237390_at | N51516 |  |  | 0.040 | 5.835 |
| 237484_at | BE501385 | C12orf69 | chromosome 12 open reading frame 69 | 0.040 | 3.055 |
| 201627_s_at | NM_005542 | INSIG1 | insulin induced gene 1 | 0.043 | 0.402 |
| 202747_s_at | NM_004867 | ITM2A | integral membrane protein 2A | 0.043 | 0.496 |
| 202834_at | NM_000029 | AGT | angiotensinogen (serpin peptidase inhibitor, clade A, member 8) | 0.043 | 4.082 |
| 202886_s_at | M65254 | PPP2R1B | protein phosphatase 2 (formerly 2A), regulatory subunit A, beta isoform | 0.043 | 3.679 |
| 204072_s_at | NM_023037 | FRY | furry homolog (Drosophila) | 0.043 | 0.413 |
| 204143_s_at | NM_017512 | ENOSF1 | enolase superfamily member 1 | 0.043 | 0.494 |
| 207339_s_at | NM_002341 | LTB | lymphotoxin beta (TNF superfamily, member 3) | 0.043 | 2.456 |
| 210513_s_at | AF091352 | VEGFA | vascular endothelial growth factor A | 0.043 | 2.092 |
| 218468_s_at | AF154054 | GREM1 | gremlin 1, cysteine knot superfamily, homolog (Xenopus laevis) | 0.043 | 0.079 |
| 219578_s_at | NM_030594 | CPEB1 | cytoplasmic polyadenylation element binding protein 1 | 0.043 | 0.405 |
| 222847_s_at | AI378406 | EGLN3 | egl nine homolog 3 (C. elegans) | 0.043 | 4.012 |
| 224451_x_at | BC006107 | ARHGAP9 | Rho GTPase activating protein 9 | 0.043 | 2.075 |
| 232136_s_at | AB051545 | CTTNBP2 | cortactin binding protein 2 | 0.043 | 0.326 |
| 232195_at | R41459 | GPR158 | G protein-coupled receptor 158 | 0.043 | 11.365 |
| 236029_at | AI283093 | FAT3 | FAT tumor suppressor homolog 3 (Drosophila) | 0.043 | 0.186 |
| 1552511_a_at | NM_020361 | CPA6 | carboxypeptidase A6 | 0.047 | 2.443 |
| 201787_at | NM_001996 | FBLN1 | fibulin 1 | 0.047 | 2.408 |
| 202082_s_at | AV748469 | SEC14L1 | SEC14-like 1 (S. cerevisiae) | 0.047 | 2.120 |
| 202219_at | NM_005629 | SLC6A8 | solute carrier family 6 (neurotransmitter transporter, creatine), member 8 | 0.047 | 2.806 |
| 203523_at | NM_002339 | LSP1 | lymphocyte-specific protein 1 | 0.047 | 2.058 |
| 205100_at | NM_005110 | GFPT2 | glutamine-fructose-6-phosphate transaminase 2 | 0.047 | 2.186 |
| 209457_at | U16996 | DUSP5 | dual specificity phosphatase 5 | 0.047 | 2.317 |
| 209542_x_at | M29644 | IGF1 | insulin-like growth factor 1 (somatomedin C) | 0.047 | 5.052 |
| 211577_s_at | M37484 | IGF1 | insulin-like growth factor 1 (somatomedin C) | 0.047 | 4.684 |
| 213552_at | W87398 | GLCE | glucuronic acid epimerase | 0.047 | 4.934 |
| 213603_s_at | BE138888 | RAC2 | ras-related C3 botulinum toxin substrate 2 (rho family, small GTP binding protein Rac2) | 0.047 | 2.079 |
| 224941_at | BF107618 | PAPPA | pregnancy-associated plasma protein A, pappalysin 1 | 0.047 | 3.288 |
| 228320_x_at | R61322 | CCDC64 | coiled-coil domain containing 64 | 0.047 | 2.562 |
| 231766_s_at | U73778 | COL12A1 | collagen, type XII, alpha 1 | 0.047 | 4.824 |
| 237177_at | AW241703 | CNTN4 | contactin 4 | 0.047 | 3.718 |
| 202794_at | NM_002194 | INPP1 | inositol polyphosphate-1-phosphatase | 0.047 | 0.383 |
| 209576_at | AL049933 | GNAI1 | guanine nucleotide binding protein (G protein), alpha inhibiting activity polypeptide 1 | 0.047 | 0.331 |
| 210375_at | X83858 | PTGER3 | prostaglandin E receptor 3 (subtype EP3) | 0.047 | 0.184 |
| 213610_s_at | BE326381 | KLHL23 | kelch-like 23 (Drosophila) | 0.047 | 0.413 |
| 215617_at | AU145711 | LOC26010 | viral DNA polymerase-transactivated protein 6 | 0.047 | 0.293 |
| 1553194_at | NM_173808 | NEGR1 | neuronal growth regulator 1 | 0.050 | 0.307 |
| 1564002_a_at | AK092103 | C6orf199 | chromosome 6 open reading frame 199 | 0.050 | 0.487 |
| 201313_at | NM_001975 | ENO2 | enolase 2 (gamma, neuronal) | 0.050 | 0.242 |
| 201626_at | BG292233 | INSIG1 | insulin induced gene 1 | 0.050 | 0.371 |
| 203929_s_at | AI056359 | MAPT | microtubule-associated protein tau | 0.050 | 3.310 |
| 204042_at | AB020707 | WASF3 | WAS protein family, member 3 | 0.050 | 2.155 |
| 205352_at | NM_005025 | SERPINI1 | serpin peptidase inhibitor, clade I (neuroserpin), member 1 | 0.050 | 2.382 |
| 205593_s_at | NM_002606 | PDE9A | phosphodiesterase 9A | 0.050 | 2.072 |
| 206401_s_at | J03778 | MAPT | microtubule-associated protein tau | 0.050 | 3.839 |
| 209083_at | U34690 | CORO1A | coronin, actin binding protein, 1A | 0.050 | 2.046 |
| 209386_at | AI346835 | TM4SF1 | transmembrane 4 L six family member 1 | 0.050 | 2.096 |
| 210095_s_at | M31159 | IGFBP3 | insulin-like growth factor binding protein 3 | 0.050 | 2.390 |
| 211538_s_at | U56725 | HSPA2 | heat shock 70kDa protein 2 | 0.050 | 2.481 |
| 212560_at | AV728268 | SORL1 | sortilin-related receptor, L(DLR class) A repeats-containing | 0.050 | 3.081 |
| 214906_x_at | AL049786 | N4BP2L1 | NEDD4 binding protein 2-like 1 | 0.050 | 0.432 |
| 217984_at | NM_003730 | RNASET2 | ribonuclease T2 | 0.050 | 2.390 |
| 219049_at | NM_018371 | CSGALNACT1 | chondroitin sulfate N-acetylgalactosaminyltransferase 1 | 0.050 | 2.012 |
| 221796_at | AA707199 | NTRK2 | neurotrophic tyrosine kinase, receptor, type 2 | 0.050 | 3.318 |
| 222862_s_at | BG169832 | AK5 | adenylate kinase 5 | 0.050 | 2.383 |
| 223475_at | AF142573 | CRISPLD1 | cysteine-rich secretory protein LCCL domain containing 1 | 0.050 | 0.378 |
| 224488_s_at | BC006262 | SPON1 | spondin 1, extracellular matrix protein | 0.050 | 2.771 |
| 224940_s_at | BF107618 | PAPPA | pregnancy-associated plasma protein A, pappalysin 1 | 0.050 | 3.319 |
| 224942_at | BG434272 | PAPPA | pregnancy-associated plasma protein A, pappalysin 1 | 0.050 | 4.235 |
| 226448_at | AI130705 | FAM89A | family with sequence similarity 89, member A | 0.050 | 0.403 |
| 229723_at | BF591040 | TAGAP | T-cell activation RhoGTPase activating protein | 0.050 | 2.026 |
| 200644_at | NM_023009 | MARCKSL1 | MARCKS-like 1 | 0.054 | 2.130 |
| 201340_s_at | AF010314 | ENC1 | ectodermal-neural cortex (with BTB-like domain) | 0.054 | 0.271 |
| 203180_at | NM_000693 | ALDH1A3 | aldehyde dehydrogenase 1 family, member A3 | 0.054 | 2.603 |
| 203928_x_at | AI870749 | MAPT | microtubule-associated protein tau | 0.054 | 3.640 |
| 205381_at | NM_005824 | LRRC17 | leucine rich repeat containing 17 | 0.054 | 0.151 |
| 206227_at | NM_003613 | CILP | cartilage intermediate layer protein, nucleotide pyrophosphohydrolase | 0.054 | 3.955 |
| 207419_s_at | NM_002872 | RAC2 | ras-related C3 botulinum toxin substrate 2 (rho family, small GTP binding protein Rac2) | 0.054 | 2.207 |
| 212143_s_at | BF340228 | IGFBP3 | insulin-like growth factor binding protein 3 | 0.054 | 2.415 |
| 212171_x_at | H95344 | VEGFA | vascular endothelial growth factor A | 0.054 | 2.299 |
| 214769_at | AF052117 | CLCN4 | chloride channel 4 | 0.054 | 2.571 |
| 218002_s_at | NM_004887 | CXCL14 | chemokine (C-X-C motif) ligand 14 | 0.054 | 16.022 |
| 219134_at | NM_022159 | ELTD1 | EGF, latrophilin and seven transmembrane domain containing 1 | 0.054 | 2.693 |
| 219310_at | NM_024893 | C20orf39 | chromosome 20 open reading frame 39 | 0.054 | 0.102 |
| 221795_at | AI346341 | NTRK2 | neurotrophic tyrosine kinase, receptor, type 2 | 0.054 | 3.727 |
| 223235_s_at | AB014737 | SMOC2 | SPARC related modular calcium binding 2 | 0.054 | 3.542 |
| 223316_at | AL136562 | CCDC3 | coiled-coil domain containing 3 | 0.054 | 5.564 |
| 225078_at | AV686514 | EMP2 | epithelial membrane protein 2 | 0.054 | 2.182 |
| 225388_at | AI928507 | TSPAN5 | tetraspanin 5 | 0.054 | 0.257 |
| 226219_at | AW575123 | ARHGAP30 | Rho GTPase activating protein 30 | 0.054 | 2.001 |
| 228128_x_at | AI110886 | PAPPA | pregnancy-associated plasma protein A, pappalysin 1 | 0.054 | 3.226 |
| 235763_at | AA001450 | SLC44A5 | solute carrier family 44, member 5 | 0.054 | 0.249 |
| 236340_at | AI769947 |  |  | 0.054 | 0.497 |
| 40148_at | U62325 | APBB2 | amyloid beta (A4) precursor protein-binding, family B, member 2 | 0.054 | 0.499 |
| 1552388_at | NM_152514 | FLJ30901 | hypothetical protein FLJ30901 | 0.058 | 2.653 |
| 1553175_s_at | AB015656 | PDE5A | phosphodiesterase 5A, cGMP-specific | 0.058 | 0.405 |
| 201981_at | AA148534 | PAPPA | pregnancy-associated plasma protein A, pappalysin 1 | 0.058 | 3.865 |
| 202724_s_at | NM_002015 | FOXO1 | forkhead box O1 | 0.058 | 2.087 |
| 203689_s_at | AI743037 | FMR1 | fragile X mental retardation 1 | 0.058 | 3.505 |
| 204529_s_at | AI961231 | TOX | thymocyte selection-associated high mobility group box | 0.058 | 0.149 |
| 205442_at | NM_021647 | MFAP3L | microfibrillar-associated protein 3-like | 0.058 | 2.029 |
| 206960_at | NM_005296 | LPAR4 | lysophosphatidic acid receptor 4 | 0.058 | 0.285 |
| 210377_at | D16350 | ACSM3 | acyl-CoA synthetase medium-chain family member 3 | 0.058 | 0.073 |
| 211178_s_at | AF038602 | PSTPIP1 | proline-serine-threonine phosphatase interacting protein 1 | 0.058 | 0.078 |
| 211494_s_at | AF157492 | SLC4A4 | solute carrier family 4, sodium bicarbonate cotransporter, member 4 | 0.058 | 0.056 |
| 214667_s_at | AK026607 | TP53I11 | tumor protein p53 inducible protein 11 | 0.058 | 2.158 |
| 220504_at | NM_007035 | KERA | keratocan | 0.058 | 2.774 |
| 222043_at | AI982754 | CLU | clusterin | 0.058 | 2.605 |
| 222570_at | AA045247 | FREQ | frequenin homolog (Drosophila) | 0.058 | 2.092 |
| 222885_at | AF205940 | EMCN | endomucin | 0.058 | 2.042 |
| 224794_s_at | AA654142 | CERCAM | cerebral endothelial cell adhesion molecule | 0.058 | 2.026 |
| 228094_at | AL048542 | AMICA1 | adhesion molecule, interacts with CXADR antigen 1 | 0.058 | 2.230 |
| 230061_at | AW338625 | TM4SF18 | transmembrane 4 L six family member 18 | 0.058 | 2.184 |
| 235108_at | BG105700 |  |  | 0.058 | 2.539 |
| 241682_at | BE873351 | KLHL23 | kelch-like 23 (Drosophila) | 0.058 | 0.398 |
| 200832_s_at | AB032261 | SCD | stearoyl-CoA desaturase (delta-9-desaturase) | 0.063 | 0.128 |
| 203185_at | NM_014737 | RASSF2 | Ras association (RalGDS/AF-6) domain family member 2 | 0.063 | 0.465 |
| 203498_at | NM_005822 | RCAN2 | regulator of calcineurin 2 | 0.063 | 0.330 |
| 204037_at | BF055366 | LPAR1 | lysophosphatidic acid receptor 1 | 0.063 | 0.229 |
| 204161_s_at | NM_014936 | ENPP4 | ectonucleotide pyrophosphatase/phosphodiesterase 4 (putative function) | 0.063 | 0.381 |
| 204475_at | NM_002421 | MMP1 | matrix metallopeptidase 1 (interstitial collagenase) | 0.063 | 2.224 |
| 204948_s_at | NM_013409 | FST | follistatin | 0.063 | 0.133 |
| 205291_at | NM_000878 | IL2RB | interleukin 2 receptor, beta | 0.063 | 2.912 |
| 208997_s_at | U82819 | UCP2 | uncoupling protein 2 (mitochondrial, proton carrier) | 0.063 | 2.700 |
| 212281_s_at | BF038366 | TMEM97 | transmembrane protein 97 | 0.063 | 3.570 |
| 213194_at | BF059159 | ROBO1 | roundabout, axon guidance receptor, homolog 1 (Drosophila) | 0.063 | 0.351 |
| 215388_s_at | X56210 | CFHR1 | complement factor H-related 1 | 0.063 | 0.448 |
| 217197_x_at | AL049785 | N4BP2L1 | NEDD4 binding protein 2-like 1 | 0.063 | 0.451 |
| 221245_s_at | NM_030804 | FZD5 | frizzled homolog 5 (Drosophila) | 0.063 | 5.738 |
| 221261_x_at | NM_030801 | MAGED4B | melanoma antigen family D, 4B | 0.063 | 0.455 |
| 222484_s_at | AF144103 | CXCL14 | chemokine (C-X-C motif) ligand 14 | 0.063 | 14.936 |
| 223611_s_at | AI738919 | LNX1 | ligand of numb-protein X 1 | 0.063 | 5.102 |
| 225627_s_at | AK024256 | CACHD1 | cache domain containing 1 | 0.063 | 0.456 |
| 227401_at | BE856748 | IL17D | interleukin 17D | 0.063 | 0.020 |
| 228127_at | BF513479 |  |  | 0.063 | 2.413 |
| 230625_s_at | AI056699 | TSPAN12 | tetraspanin 12 | 0.063 | 0.181 |
| 237833_s_at | BF062366 | SNCAIP | synuclein, alpha interacting protein | 0.063 | 0.359 |
| 44783_s_at | R61374 | HEY1 | hairy/enhancer-of-split related with YRPW motif 1 | 0.063 | 2.141 |
| 202196_s_at | NM_013253 | DKK3 | dickkopf homolog 3 (Xenopus laevis) | 0.067 | 8.244 |
| 202555_s_at | NM_005965 | MYLK | myosin light chain kinase | 0.067 | 3.574 |
| 203650_at | NM_006404 | PROCR | protein C receptor, endothelial (EPCR) | 0.067 | 0.464 |
| 206762_at | NM_002234 | KCNA5 | potassium voltage-gated channel, shaker-related subfamily, member 5 | 0.067 | 3.676 |
| 206879_s_at | NM_013982 | NRG2 | neuregulin 2 | 0.067 | 0.461 |
| 208791_at | M25915 | CLU | clusterin | 0.067 | 2.059 |
| 208792_s_at | M25915 | CLU | clusterin | 0.067 | 2.152 |
| 209168_at | AW148844 | GPM6B | glycoprotein M6B | 0.067 | 0.203 |
| 210031_at | J04132 | CD247 | CD247 molecule | 0.067 | 2.344 |
| 214247_s_at | AU148057 | DKK3 | dickkopf homolog 3 (Xenopus laevis) | 0.067 | 6.966 |
| 219778_at | NM_012082 | ZFPM2 | zinc finger protein, multitype 2 | 0.067 | 0.382 |
| 220975_s_at | NM_030968 | C1QTNF1 | C1q and tumor necrosis factor related protein 1 | 0.067 | 2.524 |
| 222450_at | AL035541 | PMEPA1 | prostate transmembrane protein, androgen induced 1 | 0.067 | 2.338 |
| 223217_s_at | BE646573 | NFKBIZ | nuclear factor of kappa light polypeptide gene enhancer in B-cells inhibitor, zeta | 0.067 | 3.488 |
| 224823_at | AA526844 | MYLK | myosin light chain kinase | 0.067 | 3.086 |
| 227530_at | BF511276 | AKAP12 | A kinase (PRKA) anchor protein 12 | 0.067 | 2.327 |
| 231157_at | BE856668 | TTLL11 | tubulin tyrosine ligase-like family, member 11 | 0.067 | 0.385 |
| 231983_at | BG471870 | C1orf69 | chromosome 1 open reading frame 69 | 0.067 | 0.210 |
| 232612_s_at | AK001690 | ATG16L1 | ATG16 autophagy related 16-like 1 (S. cerevisiae) | 0.067 | 0.380 |
| 236302_at | R40892 | PPM1E | protein phosphatase 1E (PP2C domain containing) | 0.067 | 2.128 |
| 236378_at | BF681360 | CIB4 | calcium and integrin binding family member 4 | 0.067 | 3.729 |
| 239439_at | BF963382 | AFF4 | AF4/FMR2 family, member 4 | 0.067 | 0.484 |
| 242385_at | R18374 | RORB | RAR-related orphan receptor B | 0.067 | 9.968 |
| 243946_at | AI679149 | SMOC2 | SPARC related modular calcium binding 2 | 0.067 | 4.040 |
| 1555725_a_at | AF493929 | RGS5 | regulator of G-protein signaling 5 | 0.072 | 0.308 |
| 203184_at | NM_001999 | FBN2 | fibrillin 2 | 0.072 | 0.251 |
| 204160_s_at | AW194947 | ENPP4 | ectonucleotide pyrophosphatase/phosphodiesterase 4 (putative function) | 0.072 | 0.461 |
| 223313_s_at | BC001207 | MAGED4B | melanoma antigen family D, 4B | 0.072 | 0.469 |
| 226420_at | BG261252 | EVI1 | ecotropic viral integration site 1 | 0.072 | 0.118 |
| 227692_at | AU153866 | GNAI1 | guanine nucleotide binding protein (G protein), alpha inhibiting activity polypeptide 1 | 0.072 | 0.345 |
| 201693_s_at | AV733950 | EGR1 | early growth response 1 | 0.072 | 2.855 |
| 203140_at | NM_001706 | BCL6 | B-cell CLL/lymphoma 6 | 0.072 | 2.077 |
| 206101_at | NM_001393 | ECM2 | extracellular matrix protein 2, female organ and adipocyte specific | 0.072 | 2.365 |
| 208983_s_at | M37780 | PECAM1 | platelet/endothelial cell adhesion molecule | 0.072 | 2.422 |
| 209496_at | BC000069 | RARRES2 | retinoic acid receptor responder (tazarotene induced) 2 | 0.072 | 2.251 |
| 210221_at | BC000513 | CHRNA3 | cholinergic receptor, nicotinic, alpha 3 | 0.072 | 2.806 |
| 212806_at | AL138349 | LOC100129762 | similar to KIAA0367 | 0.072 | 2.916 |
| 213030_s_at | AI688418 | PLXNA2 | plexin A2 | 0.072 | 2.044 |
| 214680_at | BF674712 | NTRK2 | neurotrophic tyrosine kinase, receptor, type 2 | 0.072 | 3.698 |
| 219014_at | NM_016619 | PLAC8 | placenta-specific 8 | 0.072 | 2.953 |
| 219308_s_at | NM_012093 | AK5 | adenylate kinase 5 | 0.072 | 2.396 |
| 227404_s_at | AI459194 | EGR1 | early growth response 1 | 0.072 | 2.081 |
| 228532_at | AW662189 | C1orf162 | chromosome 1 open reading frame 162 | 0.072 | 2.087 |
| 231478_at | AI051127 | PDE4C | phosphodiesterase 4C, cAMP-specific (phosphodiesterase E1 dunce homolog, Drosophila) | 0.072 | 2.512 |
| 1554334_a_at | BC031044 | DNAJA4 | DnaJ (Hsp40) homolog, subfamily A, member 4 | 0.078 | 2.319 |
| 1558964_at | AA334950 | FAT3 | FAT tumor suppressor homolog 3 (Drosophila) | 0.078 | 0.164 |
| 1559965_at | BC037827 |  |  | 0.078 | 0.497 |
| 202007_at | BF940043 | NID1 | nidogen 1 | 0.078 | 2.071 |
| 204105_s_at | NM_005010 | NRCAM | neuronal cell adhesion molecule | 0.078 | 0.243 |
| 204204_at | NM_001860 | SLC31A2 | solute carrier family 31 (copper transporters), member 2 | 0.078 | 2.094 |
| 208450_at | NM_006498 | LGALS2 | lectin, galactoside-binding, soluble, 2 | 0.078 | 2.762 |
| 209070_s_at | AI183997 | RGS5 | regulator of G-protein signaling 5 | 0.078 | 0.387 |
| 209890_at | AF065389 | TSPAN5 | tetraspanin 5 | 0.078 | 0.308 |
| 209955_s_at | U76833 | FAP | fibroblast activation protein, alpha | 0.078 | 2.639 |
| 210001_s_at | AB005043 | SOCS1 | suppressor of cytokine signaling 1 | 0.078 | 2.028 |
| 210739_x_at | AF069510 | SLC4A4 | solute carrier family 4, sodium bicarbonate cotransporter, member 4 | 0.078 | 0.065 |
| 212977_at | AI817041 | CXCR7 | chemokine (C-X-C motif) receptor 7 | 0.078 | 2.320 |
| 218718_at | NM_016205 | PDGFC | platelet derived growth factor C | 0.078 | 0.381 |
| 218966_at | NM_018728 | MYO5C | myosin VC | 0.078 | 2.191 |
| 219274_at | NM_012338 | TSPAN12 | tetraspanin 12 | 0.078 | 0.257 |
| 225328_at | N21643 |  |  | 0.078 | 2.456 |
| 226086_at | AB037848 | SYT13 | synaptotagmin XIII | 0.078 | 6.793 |
| 230030_at | AI767756 | HS6ST2 | heparan sulfate 6-O-sulfotransferase 2 | 0.078 | 2.266 |
| 230258_at | AI277316 | GLIS3 | GLIS family zinc finger 3 | 0.078 | 2.191 |
| 1552767_a_at | NM_147174 | HS6ST2 | heparan sulfate 6-O-sulfotransferase 2 | 0.083 | 2.158 |
| 1559966_a_at | BC037827 |  |  | 0.083 | 0.477 |
| 201341_at | NM_003633 | ENC1 | ectodermal-neural cortex (with BTB-like domain) | 0.083 | 0.277 |
| 204864_s_at | NM_002184 | IL6ST | interleukin 6 signal transducer (gp130, oncostatin M receptor) | 0.083 | 0.440 |
| 204897_at | AA897516 | PTGER4 | prostaglandin E receptor 4 (subtype EP4) | 0.083 | 2.929 |
| 206791_s_at | BF511742 | PDE4C | phosphodiesterase 4C, cAMP-specific (phosphodiesterase E1 dunce homolog, Drosophila) | 0.083 | 2.442 |
| 209071_s_at | AF159570 | RGS5 | regulator of G-protein signaling 5 | 0.083 | 0.452 |
| 209387_s_at | M90657 | TM4SF1 | transmembrane 4 L six family member 1 | 0.083 | 2.026 |
| 213010_at | AI088622 | PRKCDBP | protein kinase C, delta binding protein | 0.083 | 0.460 |
| 213419_at | U62325 | APBB2 | amyloid beta (A4) precursor protein-binding, family B, member 2 | 0.083 | 0.473 |
| 215245_x_at | AA830884 | FMR1 | fragile X mental retardation 1 | 0.083 | 3.021 |
| 216044_x_at | AK027146 | FAM69A | family with sequence similarity 69, member A | 0.083 | 0.372 |
| 220432_s_at | NM_016593 | CYP39A1 | cytochrome P450, family 39, subfamily A, polypeptide 1 | 0.083 | 0.474 |
| 222258_s_at | AF015043 | SH3BP4 | SH3-domain binding protein 4 | 0.083 | 2.552 |
| 225387_at | AA059445 | TSPAN5 | tetraspanin 5 | 0.083 | 0.278 |
| 228640_at | BE644809 | PCDH7 | protocadherin 7 | 0.083 | 0.228 |
| 231887_s_at | AB033100 | KIAA1274 | KIAA1274 | 0.083 | 0.499 |
| 243357_at | AA115106 | NEGR1 | neuronal growth regulator 1 | 0.083 | 0.413 |
| 208982_at | AW574504 | PECAM1 | platelet/endothelial cell adhesion molecule | 0.089 | 2.507 |
| 209170_s_at | AF016004 | GPM6B | glycoprotein M6B | 0.089 | 0.212 |
| 209398_at | BC002649 | HIST1H1C | histone cluster 1, H1c | 0.089 | 0.478 |
| 211587_x_at | M37981 | CHRNA3 | cholinergic receptor, nicotinic, alpha 3 | 0.089 | 2.472 |
| 213050_at | AA594937 | COBL | cordon-bleu homolog (mouse) | 0.089 | 2.372 |
| 214617_at | AI445650 | PRF1 | perforin 1 (pore forming protein) | 0.089 | 2.389 |
| 216834_at | S59049 | RGS1 | regulator of G-protein signaling 1 | 0.089 | 2.290 |
| 221884_at | BE466525 | EVI1 | ecotropic viral integration site 1 | 0.089 | 0.150 |
| 229854_at | AW614056 | OBSCN | obscurin, cytoskeletal calmodulin and titin-interacting RhoGEF | 0.089 | 0.471 |
| 232267_at | AL162032 | GPR133 | G protein-coupled receptor 133 | 0.089 | 2.267 |
| 235171_at | AI354636 |  |  | 0.089 | 0.204 |
| 241729_at | AW173080 | DOK6 | docking protein 6 | 0.089 | 0.493 |
| 47069_at | AA533284 | PRR5 | proline rich 5 (renal) | 0.089 | 2.193 |
| 202274_at | NM_001615 | ACTG2 | actin, gamma 2, smooth muscle, enteric | 0.096 | 4.162 |
| 203065_s_at | NM_001753 | CAV1 | caveolin 1, caveolae protein, 22kDa | 0.096 | 2.075 |
| 204036_at | AW269335 | LPAR1 | lysophosphatidic acid receptor 1 | 0.096 | 0.262 |
| 204834_at | NM_006682 | FGL2 | fibrinogen-like 2 | 0.096 | 0.494 |
| 211813_x_at | AF138303 | DCN | decorin | 0.096 | 3.596 |
| 212097_at | AU147399 | CAV1 | caveolin 1, caveolae protein, 22kDa | 0.096 | 2.080 |
| 213435_at | AB028957 | SATB2 | SATB homeobox 2 | 0.096 | 0.165 |
| 217739_s_at | NM_005746 | NAMPT | nicotinamide phosphoribosyltransferase | 0.096 | 2.386 |
| 217979_at | NM_014399 | TSPAN13 | tetraspanin 13 | 0.096 | 2.416 |
| 217995_at | NM_021199 | SQRDL | sulfide quinone reductase-like (yeast) | 0.096 | 0.441 |
| 228121_at | AU145950 |  |  | 0.096 | 2.061 |
| 228158_at | AI623211 | LOC645166 | similar to lymphocyte-specific protein 1 | 0.096 | 0.481 |
| 231856_at | AB033070 | KIAA1244 | KIAA1244 | 0.096 | 0.289 |

Supplementary table 4

1. Gene ontology terms overrepresented in probesets differentiating samples according to mutation status

| Id | GO_description | p.values | pa.values |
| --- | --- | --- | --- |
| GO:0001568 | blood vessel development | 1.81E-06 | 0.001379 |
| GO:0001525 | angiogenesis | 8.40E-06 | 0.003196 |
| GO:0016337 | cell-cell adhesion | 3.02E-05 | 0.007668 |
| GO:0009887 | organ morphogenesis | 6.05E-05 | 0.011502 |
| GO:0007275 | multicellular organismal development | 0.000101 | 0.015343 |
| GO:0007204 | elevation of cytosolic calcium ion concentration | 0.000334 | 0.038139 |
| GO:0007169 | transmembrane receptor protein tyrosine kinase signaling pathway | 0.000351 | 0.038139 |
| GO:0048513 | organ development | 0.000488 | 0.042541 |
| GO:0048869 | cellular developmental process | 0.000503 | 0.042541 |

1. Gene ontology terms overrepresented in probesets differentiating samples according to expression of KIT

| Ids | GO_description | p.values | pa.values |
| --- | --- | --- | --- |
| GO:0048731 | system development | 3.45E-06 | 0.005556 |
| GO:0001568 | blood vessel development | 1.12E-05 | 0.008982 |
| GO:0007155 | cell adhesion | 4.17E-05 | 0.018507 |
| GO:0007165 | signal transduction | 4.60E-05 | 0.018507 |
| GO:0007186 | G-protein coupled receptor protein signaling pathway | 6.87E-05 | 0.022111 |
| GO:0008037 | cell recognition | 0.000135 | 0.036188 |
| GO:0009887 | organ morphogenesis | 0.000158 | 0.036248 |

Supplementary table 5

Probesets with expression changed at least 2 fold between samples with low and high KIT expression annotated to selected GO terms

1. GO:0007186 - G-protein interaction

| Probeset | GenBank | Symbol | Description | adj.pval | LOW | HIGH | fc |
| --- | --- | --- | --- | --- | --- | --- | --- |
| 209392_at | L35594 | ENPP2 | ectonucleotide pyrophosphatase/phosphodiesterase 2 | 0.016 | 5637.01 | 2649.72 | 2.127 |
| 204995_at | AL567411 | CDK5R1 | cyclin-dependent kinase 5, regulatory subunit 1 (p35) | 0.016 | 107.23 | 26.88 | 3.989 |
| 203823_at | NM_021106 | RGS3 | regulator of G-protein signaling 3 | 0.016 | 497.70 | 209.49 | 2.376 |
| 222834_s_at | N32508 | GNG12 | Guanine nucleotide binding protein (G protein), gamma 12 | 0.016 | 216.08 | 509.44 | 0.424 |
| 205111_s_at | NM_016341 | PLCE1 | phospholipase C, epsilon 1 | 0.017 | 397.18 | 1126.13 | 0.353 |
| 205112_at | NM_016341 | PLCE1 | phospholipase C, epsilon 1 | 0.017 | 537.23 | 1570.44 | 0.342 |
| 210839_s_at | D45421 | ENPP2 | ectonucleotide pyrophosphatase/phosphodiesterase 2 | 0.017 | 1601.73 | 654.52 | 2.447 |
| 1555240_s_at | AF493879 | GNG12 | Guanine nucleotide binding protein (G protein), gamma 12 | 0.017 | 127.95 | 335.69 | 0.381 |
| 210381_s_at | BC000740 | CCKBR | cholecystokinin B receptor | 0.017 | 969.42 | 4747.14 | 0.204 |
| 217057_s_at | AF107846 | GNAS | GNAS complex locus | 0.017 | 714.63 | 65.58 | 10.897 |
| 203632_s_at | NM_016235 | GPRC5B | G protein-coupled receptor, family C, group 5, member B | 0.019 | 117.49 | 43.79 | 2.683 |
| 210831_s_at | L27489 | PTGER3 | prostaglandin E receptor 3 (subtype EP3) | 0.020 | 95.16 | 483.69 | 0.197 |
| 213792_s_at | AA485908 | INSR | insulin receptor | 0.020 | 370.34 | 180.52 | 2.052 |
| 204639_at | NM_000022 | ADA | adenosine deaminase | 0.021 | 502.48 | 196.49 | 2.557 |
| 206187_at | NM_000960 | PTGIR | prostaglandin I2 (prostacyclin) receptor (IP) | 0.021 | 233.39 | 84.11 | 2.775 |
| 216598_s_at | S69738 | CCL2 | chemokine (C-C motif) ligand 2 | 0.021 | 872.57 | 306.68 | 2.845 |
| 216705_s_at | X02189 | ADA | adenosine deaminase | 0.021 | 221.98 | 85.38 | 2.600 |
| 210374_x_at | D38300 | PTGER3 | prostaglandin E receptor 3 (subtype EP3) | 0.021 | 53.13 | 281.29 | 0.189 |
| 227415_at | BF109303 | DGKH | diacylglycerol kinase, eta | 0.021 | 144.93 | 507.14 | 0.286 |
| 210832_x_at | D38298 | PTGER3 | prostaglandin E receptor 3 (subtype EP3) | 0.027 | 55.49 | 296.94 | 0.187 |
| 210833_at | AL031429 | PTGER3 | prostaglandin E receptor 3 (subtype EP3) | 0.029 | 77.23 | 535.80 | 0.144 |
| 204396_s_at | NM_005308 | GRK5 | G protein-coupled receptor kinase 5 | 0.032 | 745.66 | 327.70 | 2.275 |
| 205651_x_at | NM_007023 | RAPGEF4 | Rap guanine nucleotide exchange factor (GEF) 4 | 0.032 | 211.18 | 44.19 | 4.779 |
| 213933_at | AW242315 | PTGER3 | prostaglandin E receptor 3 (subtype EP3) | 0.040 | 626.53 | 2587.15 | 0.242 |
| 202834_at | NM_000029 | AGT | angiotensinogen (serpin peptidase inhibitor, clade A, member 8) | 0.043 | 682.18 | 167.10 | 4.082 |
| 232195_at | R41459 | GPR158 | G protein-coupled receptor 158 | 0.043 | 733.77 | 64.56 | 11.365 |
| 209576_at | AL049933 | GNAI1 | guanine nucleotide binding protein (G protein), alpha inhibiting activity polypeptide 1 | 0.047 | 188.68 | 570.32 | 0.331 |
| 210375_at | X83858 | PTGER3 | prostaglandin E receptor 3 (subtype EP3) | 0.047 | 58.23 | 316.67 | 0.184 |
| 219134_at | NM_022159 | ELTD1 | EGF, latrophilin and seven transmembrane domain containing 1 | 0.054 | 189.71 | 70.44 | 2.693 |
| 206960_at | NM_005296 | LPAR4 | lysophosphatidic acid receptor 4 | 0.058 | 62.98 | 220.84 | 0.285 |
| 204037_at | BF055366 | LPAR1 | lysophosphatidic acid receptor 1 | 0.063 | 65.18 | 284.84 | 0.229 |
| 221245_s_at | NM_030804 | FZD5 | frizzled homolog 5 (Drosophila) | 0.063 | 234.81 | 40.92 | 5.738 |
| 227530_at | BF511276 | AKAP12 | A kinase (PRKA) anchor protein 12 | 0.067 | 219.27 | 94.22 | 2.327 |
| 1555725_a_at | AF493929 | RGS5 | regulator of G-protein signaling 5 | 0.072 | 2625.94 | 8520.53 | 0.308 |
| 227692_at | AU153866 | GNAI1 | guanine nucleotide binding protein (G protein), alpha inhibiting activity polypeptide 1 | 0.072 | 221.15 | 641.46 | 0.345 |
| 209070_s_at | AI183997 | RGS5 | regulator of G-protein signaling 5 | 0.078 | 4249.85 | 10979.77 | 0.387 |
| 212977_at | AI817041 | CXCR7 | chemokine (C-X-C motif) receptor 7 | 0.078 | 6076.48 | 2619.58 | 2.320 |
| 204897_at | AA897516 | PTGER4 | prostaglandin E receptor 4 (subtype EP4) | 0.083 | 888.56 | 303.36 | 2.929 |
| 209071_s_at | AF159570 | RGS5 | regulator of G-protein signaling 5 | 0.083 | 8455.00 | 18702.42 | 0.452 |
| 216834_at | S59049 | RGS1 | regulator of G-protein signaling 1 | 0.089 | 207.46 | 90.58 | 2.290 |
| 232267_at | AL162032 | GPR133 | G protein-coupled receptor 133 | 0.089 | 564.77 | 249.15 | 2.267 |
| 204036_at | AW269335 | LPAR1 | lysophosphatidic acid receptor 1 | 0.096 | 93.05 | 354.63 | 0.262 |

1. GO:0007268 - synaptic transmission

| Probeset | GenBank | Symbol | Description | adj.pval | LOW | HIGH | fc |
| --- | --- | --- | --- | --- | --- | --- | --- |
| 203231_s_at | AW235612 | ATXN1 | ataxin 1 | 0.016 | 136.56 | 417.03 | 0.327 |
| 205902_at | AJ251016 | KCNN3 | Potassium intermediate/small conductance calcium-activated channel, subfamily N, member 3 | 0.016 | 990.40 | 249.78 | 3.965 |
| 205903_s_at | NM_002249 | KCNN3 | Potassium intermediate/small conductance calcium-activated channel, subfamily N, member 3 | 0.016 | 710.26 | 179.05 | 3.967 |
| 209234_at | BF939474 | KIF1B | kinesin family member 1B | 0.016 | 61.91 | 137.32 | 0.451 |
| 205893_at | NM_014932 | NLGN1 | neuroligin 1 | 0.016 | 54.10 | 465.55 | 0.116 |
| 231361_at | AI912122 | NLGN1 | neuroligin 1 | 0.016 | 27.54 | 232.42 | 0.118 |
| 228776_at | AA430014 | GJC1 | gap junction protein, gamma 1, 45kDa | 0.017 | 632.04 | 122.14 | 5.175 |
| 203232_s_at | NM_000332 | ATXN1 | ataxin 1 | 0.022 | 643.83 | 1336.40 | 0.482 |
| 210198_s_at | BC002665 | PLP1 | proteolipid protein 1 | 0.024 | 1049.86 | 32.67 | 32.131 |
| 230087_at | AI823645 | PRIMA1 | proline rich membrane anchor 1 | 0.029 | 619.04 | 97.33 | 6.360 |
| 230109_at | AI638433 | PDE7B | phosphodiesterase 7B | 0.029 | 349.21 | 77.19 | 4.524 |
| 230849_at | N64750 | KCNA1 | potassium voltage-gated channel, shaker-related subfamily, member 1 (episodic ataxia with myokymia) | 0.029 | 866.63 | 294.84 | 2.939 |
| 229084_at | R42166 | CNTN4 | contactin 4 | 0.034 | 635.26 | 166.17 | 3.823 |
| 205952_at | NM_002246 | KCNK3 | potassium channel, subfamily K, member 3 | 0.037 | 11770.79 | 5272.29 | 2.233 |
| 232136_s_at | AB051545 | CTTNBP2 | Cortactin binding protein 2 | 0.043 | 52.11 | 160.08 | 0.326 |
| 237177_at | AW241703 | CNTN4 | contactin 4 | 0.047 | 120.30 | 32.36 | 3.718 |
| 237833_s_at | BF062366 | SNCAIP | synuclein, alpha interacting protein | 0.063 | 129.24 | 359.96 | 0.359 |
| 210221_at | BC000513 | CHRNA3 | cholinergic receptor, nicotinic, alpha 3 | 0.072 | 2205.51 | 785.95 | 2.806 |
| 211587_x_at | M37981 | CHRNA3 | cholinergic receptor, nicotinic, alpha 3 | 0.089 | 301.36 | 121.89 | 2.472 |

1. GO:0001568 blood vessel development

| Probe | GenBank | Symbol | Description | adj.pval | LOW | HIGH | fc |
| --- | --- | --- | --- | --- | --- | --- | --- |
| 200921_s_at | NM_001731 | BTG1 | B-cell translocation gene 1, anti-proliferative | 0.016 | 6595.21 | 3254.93 | 2.026 |
| 200920_s_at | AL535380 | BTG1 | B-cell translocation gene 1, anti-proliferative | 0.016 | 6455.13 | 2827.22 | 2.283 |
| 212226_s_at | AA628586 | PPAP2B | phosphatidic acid phosphatase type 2B | 0.016 | 7126.69 | 2189.09 | 3.256 |
| 209355_s_at | AB000889 | PPAP2B | phosphatidic acid phosphatase type 2B | 0.016 | 5829.21 | 1686.69 | 3.456 |
| 212230_at | AV725664 | PPAP2B | phosphatidic acid phosphatase type 2B | 0.016 | 3312.03 | 980.26 | 3.379 |
| 225263_at | BC001196 | HS6ST1 | heparan sulfate 6-O-sulfotransferase 1 | 0.016 | 512.12 | 165.09 | 3.102 |
| 200878_at | AF052094 | EPAS1 | endothelial PAS domain protein 1 | 0.016 | 1159.38 | 428.99 | 2.703 |
| 226028_at | AA156022 | ROBO4 | roundabout homolog 4, magic roundabout (Drosophila) | 0.016 | 240.30 | 67.80 | 3.544 |
| 228776_at | AA430014 | GJC1 | gap junction protein, gamma 1, 45kDa | 0.017 | 632.04 | 122.14 | 5.175 |
| 203934_at | NM_002253 | KDR | kinase insert domain receptor (a type III receptor tyrosine kinase) | 0.021 | 260.29 | 92.30 | 2.820 |
| 201791_s_at | NM_001360 | DHCR7 | 7-dehydrocholesterol reductase | 0.024 | 43.87 | 95.53 | 0.459 |
| 210512_s_at | AF022375 | VEGFA | vascular endothelial growth factor A | 0.025 | 1000.79 | 324.10 | 3.088 |
| 222033_s_at | AA058828 | FLT1 | fms-related tyrosine kinase 1 (vascular endothelial growth factor/vascular permeability factor receptor) | 0.027 | 206.33 | 77.27 | 2.670 |
| 208850_s_at | AL558479 | THY1 | Thy-1 cell surface antigen | 0.040 | 1761.33 | 872.64 | 2.018 |
| 213869_x_at | AA218868 | THY1 | Thy-1 cell surface antigen | 0.040 | 1537.31 | 762.92 | 2.015 |
| 202834_at | NM_000029 | AGT | angiotensinogen (serpin peptidase inhibitor, clade A, member 8) | 0.043 | 682.18 | 167.10 | 4.082 |
| 210513_s_at | AF091352 | VEGFA | vascular endothelial growth factor A | 0.043 | 148.94 | 71.20 | 2.092 |
| 212171_x_at | H95344 | VEGFA | vascular endothelial growth factor A | 0.054 | 273.22 | 118.84 | 2.299 |
| 202724_s_at | NM_002015 | FOXO1 | forkhead box O1 | 0.058 | 317.72 | 152.23 | 2.087 |
| 222885_at | AF205940 | EMCN | Endomucin | 0.058 | 210.61 | 103.16 | 2.042 |
| 44783_s_at | R61374 | HEY1 | hairy/enhancer-of-split related with YRPW motif 1 | 0.063 | 195.47 | 91.28 | 2.141 |
| 203065_s_at | NM_001753 | CAV1 | caveolin 1, caveolae protein, 22kDa | 0.096 | 784.81 | 378.26 | 2.075 |
| 212097_at | AU147399 | CAV1 | caveolin 1, caveolae protein, 22kDa | 0.096 | 2332.28 | 1121.29 | 2.080 |

Supplementary table 6

Interactomes of KIT and PDGFRA receptors assembled from literature and protein interaction databases (data sources: Bg – BioGrid, B – BOND database, H – HPRD, P – PubMed). Common proteins are highlighted in yellow.

| **KIT** | **Source database** | **PDGFR** | **Source database** |
| --- | --- | --- | --- |
| CRK | P | CRK | Bg, B, H |
| GRB10 | Bg, H | GRB10 | Bg, H |
| GRB2 | Bg, B, H | GRB2 | Bg, B, H |
| PIK3R1 | Bg, B, H | PIK3R1 | Bg, B, H |
| PIK3R2 | Bg, B, H | PIK3R2 | Bg, B, H |
| PLCG1 | Bg, B, H | PLCG1 | Bg, B, H |
| PTPN11 | Bg, H | PTPN11 | Bg, B, H |
| PTPN6 | Bg, H | PTPN6 | Bg, H |
| SH3KBP1 | Bg | SH3KBP1 | Bg, H |
| SHC | P | SHC | Bg, B, H |
| SOCS1 | Bg, B, H | SOCS1 | Bg, B, H, P |
| SRC | P | SRC | B |
| SYP | B | SYP | Bg, B |
| BCR | Bg | BAG1 | H |
| BRDG1 | B, H | CAV1 | Bg |
| CBLB | Bg | CAV3 | Bg |
| CD63 | H | c-Cbl | B |
| CD81 | Bg, H | COPA | Bg, H |
| CD9 | Bg, H | COPB1 | H |
| CLTC | Bg, H | EGFR | Bg, H |
| CRKL | Bg, H | EIF2AK2 | Bg |
| DOK1 | Bg, H | EPHB1 | B |
| EPOR | B, H | FYN | H |
| GATA-1 | P | GRB14 | Bg, H |
| GRAP | Bg, H | ITGB3 | Bg |
| GRAP2 | B, H | KRTAP4-12 | Bg |
| GRB7 | Bg, H | Myc | P |
| HP1-gamma | B | NCK1 | Bg, B, H |
| INPP5D | Bg | NCK2 | Bg, B, H |
| JAK2 | Bg, H | PDAP1 | B |
| KITLG | Bg, H | PDGFB | B |
| LCK | Bg, H | PDGF-BB | B |
| LYN | Bg, H | PDGF-BB/PDGF-(beta)-R/ATP | B |
| MATK | Bg, H | PDGFD | H |
| MPDZ | Bg, H | PDGFRA | Bg |
| PI3K | P | PDGFRB | Bg |
| PTPRO | Bg, H | PIK3R3 | B |
| RFC1 | Bg | PLAUR | B |
| SH2B2 | H | Prdx2 | B |
| SPRED1 | H | PTK2 | H |
| SPRED2 | H | RAF1 | Bg, H |
| STAT1 | Bg, B, H | RASA1 | Bg, B, H |
| TEC | Bg, H | S1PR1 | Bg, H |
| YES1 | Bg | SH2BPSM1 | B |
|  |  | Shb | B |
|  |  | SLC9A3R1 | Bg, H |
|  |  | SNX1 | Bg, H |
|  |  | SNX2 | Bg |
|  |  | SNX4 | Bg |
|  |  | VAV1 | Bg |
|  |  | VAV2 | Bg |
|  |  | VAV3 | Bg |
