# Supplementary figures and images for: Functional features of gene expression profiles differentiating gastrointestinal stromal tumours according to KIT mutations and expression
Source: BMC Cancer. 2009 Nov 27;9:413. doi: 10.1186/1471-2407-9-413 (PMC2794290; doi:10.1186/1471-2407-9-413)

Supplementary  
Fig.1

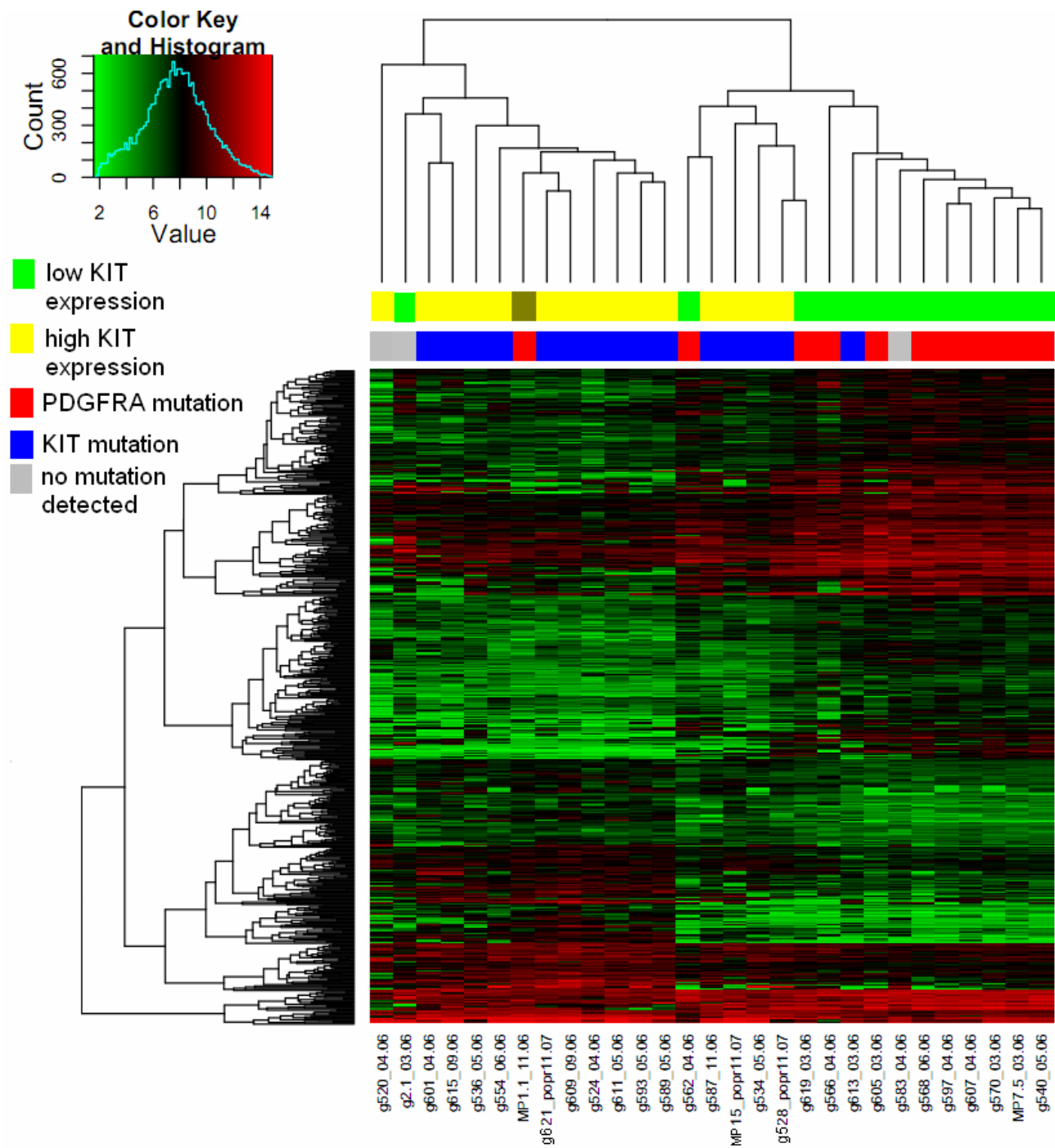

Supplement: Additional file 2 — Supplementary Figure S1. Unsupervised hierarchical clustering for the selection of differentially expressed genes in GIST tumours according to KIT mutation. Across the top, individual tumour samples are arrayed in a column (upper: green - low KIT expression/high PDGFRA expression; yellow- high KIT expression/low PDGFRA expression; dark gray- high KIT/PDGFRA expression; lower: blue - KIT mutation; red - PDGFRA mutation; gray- no mutation found); on the left side, 680 individual probe sets differentiating tumours in accordance with the KIT expression are shown in rows. The colour in each cell reflects the level of expression of the corresponding probe set in the corresponding array sample relative to its mean level of expression estimated for the entire set of samples. Red indicates expression levels greater than the mean, and green indicates lower than the mean. [file 1471-2407-9-413-S2.PDF]
